# Early-stage extraction of lithium from LCO cathodes *via* sucrose-assisted reductive roasting

**DOI:** 10.1039/d5ra08726k

**Published:** 2026-01-02

**Authors:** Martin Jantson, Kerli Liivand, Valdek Mikli

**Affiliations:** a National Institute of Chemical Physics and Biophysics 12618 Tallinn Estonia kerli.liivand@kbfi.ee; b Department of Materials and Environmental Technology, Tallinn University of Technology 19086 Tallinn Estonia

## Abstract

The growing demand for lithium-ion batteries (LIBs) and consequent increase in end-of-life LIBs raises concerns over battery material supply security and safe management of hazardous waste. Recycling of LIBs can help address both problems by utilizing waste to recover critical materials. Compared to other cathode metals like Co and Ni, Li recovery rates remain relatively low in the industry and new strategies must be adopted to reach recovery rate targets of 80% or higher by 2032 set by the European Commission. This research explores the use of food-grade sucrose as a reductant to enable selective Li recovery through reductive roasting followed by water leaching. A Li leaching efficiency of 90.1% was achieved under optimized roasting conditions (500 °C for 60 minutes with 15 wt% sucrose dosage) indentified through a systematic study of roasting parameters. Lithium extraction was further improved to 94.5% by introducing additional pre-treatment steps to remove F and Al from the cathode material, which were found to form insoluble compounds with lithium, LiAlO_2_ and LiF, during roasting. The Li leaching efficiency was shown to be highly dependent on the roasting temperature and sucrose dosage, while showing low sensitivity to roasting time due to the fast reduction mechanism and pyrolysis of sucrose. Notably, the roasting time could be reduced to below 10 minutes with minor impact (3.6%) on Li leaching efficiency, significantly lowering the energy demand for the process.

## Abstract 2

This research explores the use of food-grade sucrose as a reductant to enable selective Li recovery through reductive roasting followed by water leaching.

## Graphical abstract

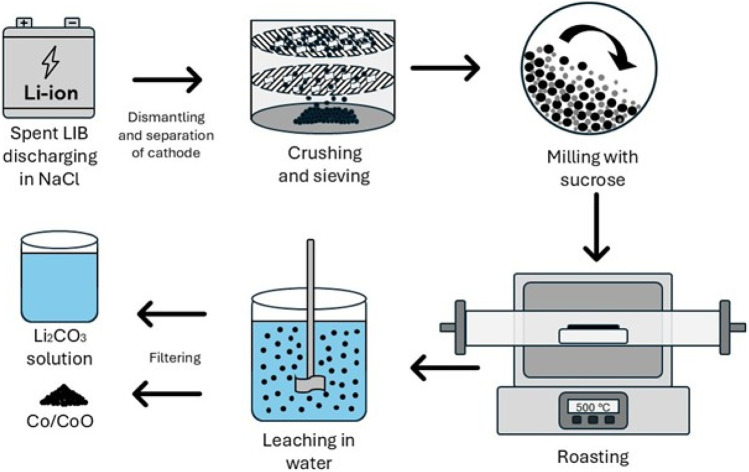

## Introduction

Lithium-ion batteries (LIBs) have become the predominant choice for energy storage solutions thanks to their relatively high energy density, low self-discharge, no memory effects, and good cycle life compared to other alternatives like lead-acid batteries and nickel metal hydride batteries.^1–4^ Current strategies for decarbonization and electrification are heavily reliant on LIBs,^5^ which has led the demand to increase from 30 GWh in 2011 to 492 GWh in 2021 (ref. 1) with automakers announcing around $1.2 trillion in investments to increase battery production capacity to almost 6 TWh by 2030.^6^ This massive increase of new LIBs will eventually lead to a higher number of spent LIBs as well, which will need to be collected and recycled efficiently to help lower the dependency on mined resources^7^ and to reduce the environmental impacts this hazardous waste could cause.^8^

More than half of the total cost of LIBs is attributed to material expenses,^8^ with the majority of these materials categorised as critical and strategic raw materials according to the European Commission. These include Li, Co, Ni, Mn, P, and bauxite (Al) in the cathode, and graphite and Cu in the anode, which could see shortages in the near future due to the increased demand from road transport electrification and limitations in domestic supply.^9^ As a result, battery manufacturers have been developing cheaper chemistries by substituting scarce and expensive materials, for example, lowering Co concentrations in favour of Ni in LiNi_1−*x*−*y*_Mn_*x*_Co_*y*_O_2_ (NMC) chemistries from NMC111 to NMC811,^1,10^ or opting for Co and Ni free chemistries like LiFePO_4_ (LFP), where high energy densities are not the highest priority while also providing greater safety.^1^ Despite attempts to reduce dependence on critical raw materials, substituting Li has proven challenging. Many emerging battery technologies like Li–sulphur, Li–metal, Li–air, and solid state batteries exhibit a potentially higher dependence on lithium compared to Li-ion batteries making it particularly susceptible to face supply shortages in the near future.^1^ Research into Na- and K-ion batteries is also gaining popularity in both academia and industry,^11^ for example the Na-ion battery developed by LIB manufacturing giant Contemporary Amperex Technology Co. Limited (CATL, China). When these novel Na and K technologies will be mature enough to hit the market and how large of an adaption they will have is unsure, as they will most likely substitute LIBs only in applications where gravimetric and volumetric energy density requirements are lower, like short distance EVs or stationary storage.^4,12^

Industrially, LIBs are commonly recycled using hydrometallurgical methods or a combination of pyrometallurgical and hydrometallurgical processes. In pyrometallurgy, the spent LIBs are typically smelted at very high temperatures (1200–1500 °C)^13–15^ to form an alloy containing Ni, Co, and Cu. This alloy can further undergo hydrometallurgical treatment to recover the metals separately. The advantages of this method are its versatility to treat a wide range of different cathode chemistries, there are no or less stringent requirements for pre-treatment, and higher recycling capacities can be achieved when compared to hydrometallurgy. The main drawbacks with this method come from the loss of various materials, due to being used as fuel during the heat treatment – graphite, polymers, and other carbon-based materials – or lost in slag – Li, Mn, and Al. Other problems include high energy consumption and CO_2_ emissions.^16,17^ Although Li recovery has not been traditionally associated with pyrometallurgy, recovery of Li is possible through leaching of flue dust and/or slag.^17^ In hydrometallurgy, the metals are dissolved into an aqueous phase using a leaching agent and a reductant.^16^ The dissolved metals can be selectively separated by using solvent extraction, ion-exchange, or chromatographic separation,^18–20^ and finally recovered by using precipitation or evaporative crystallisation.^21^ This has the benefits of achieving high recovery efficiencies and purities and being theoretically able to recover all metals present in LIBs. The main disadvantages come from being less flexible on the different types of cathode chemistries that can be recycled together in a given process and needing additional pretreatment steps, such as discharging/deactivation, comminution, and separation to minimize the amount of impurities and to acquire black mass which mostly consists of the anode and cathode active materials.^22^ Li is also typically recovered at the last steps of the pathway, which results in significant material losses of recovered material. Additional cons also include high chemical use and wastewater production and lower capacities compared to pyrometallurgy.^16,23^

New regulations in the EU will require recyclers to recover at least 50% of lithium by 2028 and 80% by 2032.^24^ To increase the Li recovery efficiency during recycling, one of the promising strategies is to recover Li in early stage of the process. This can be achieved by applying thermal treatment to decompose or reduce the cathode active material by converting Li into water-soluble salts, while the other metals remain as solid oxides or reduced metals.^17^ Different roasting processes can be applied for this: carbothermic reduction,^25^ thermite reduction,^26^ nitration roasting,^27^ chlorine roasting,^28^ hydrogen roasting,^29^ and sulfation roasting.^30^ Compared to traditional pyrometallurgical methods like smelting, roasting is done at a much lower temperature (300–900 °C), however, it can still produce harmful emissions like Cl_2_, NO_*x*_, SO_*x*_, CO, CO_2_ and HF, which can damage the equipment and require further treatment to reduce their environmental impact.^17,29^ Carbothermal reduction has received wide attention across the literature due to the nature of being able to use graphite that is already typically present in battery waste. However, carbothermal roasting systems typically require higher temperatures (650 °C and above) or extended roasting durations (several hours) compared to nitrate and hydrogen roasting.^26,29,31^ This process can also lead to the loss of graphite and CO_2_ emissions if graphite is used as a reductive agent. In contrast, hydrogen roasting is considered a promising alternative due to lower roasting temperatures (500 °C and below) and shorter roasting times (1 hour or less). Additionally, the primary emission is water vapor (H_2_O), and Li can be recovered as LiOH, which has a significantly higher solubility in water than Li_2_CO_3_ (119.2 g L^−1^*vs.* 13.3 g L^−1^ at 25 °C, respectively). Although hydrogen is considered a highly promising reductant in various pyrometallurgical heat treatments, its application at industrial or pilot scale remains limited due to challenges associated with large-scale pure hydrogen production.^32^

The use of organic compounds (polysaccharides, biomass, plastics, *etc.*) as reducing agents in the roasting process has attracted growing interest, as they can serve as sources of both gaseous reductive products such as H_2_, CO, CH_4_, and solid carbon.^17^ Yiming Lai *et al.* demonstrated Li recovery of 88.7% by using wheat straw biomass to reduce LiCoO_2_ (LCO) into Co and Li_2_CO_3_ at 800 °C with 10 minutes roasting time.^33^ In another study, by Fengyin Zhou *et al.*, pine sawdust biomass was used to treat LCO cathode at 600 °C for 120 minutes, achieving a Li recovery rate of 94.33% with an LCO to pine sawdust ratio of 1 : 1.^34^ Even higher Li extraction rates under milder roasting conditions were reported by Baichao Zhang *et al.*, who used glucose as a reducing agent for LCO, achieving a Li leaching efficiency of 97% after roasting at 550 °C for 60 minutes.^35^ In addition to C and H_2_, intermediate products produced during the pyrolysis of organic compounds can also promote reduction, further increasing the efficiency of the process.^35^ A simplification of the possible reactions occurring during the roasting of LCO while using organic compounds are the following:^25,31,35–39^

1 2LiCoO_2_ + C ↔ Li_2_O + 2CoO + CO(g)

2 4LiCoO_2_ + C ↔ 2Li_2_O + 4CoO + CO_2_(g)

3 2LiCoO_2_ + CO(g) ↔ Li_2_O + 2CoO + CO_2_(g)

4 2CoO + C ↔ 2Co + CO_2_(g)

5 CoO + C ↔ Co + CO(g)

6 CoO + CO(g) ↔ Co + CO_2_(g)

7 Li_2_O + CO_2_(g) ↔ Li_2_CO_3_

8 2LiCoO_2_ + H_2_(g) ↔ 2LiOH + 2CoO

9 CoO + H_2_(g) ↔ Co + H_2_O

10 2LiOH + CO_2_(g) ↔ Li_2_CO_3_ + H_2_O

The presence of various reducing agents can potentially lower required heating temperature and/or duration, which will result in reduced energy requirements. In addition to that, other side-products like biochar and -oil will also be produced from organics pyrolysis. However, using organics such as biowaste or plastics as a reductant, depending on their origin or composition, can introduce new impurities/additives to the system which could affect the final yields and purities of the recovered metals.^17^

This study explores the feasibility of employing food-grade sucrose as a cost-effective reductant for roasting. Sucrose decomposes during the heating into H_2_, CO, and CH_4_,^40^ providing both the carbon and hydrogen required for the reduction of the LCO. In addition to that, transition metals and CO_2_-reactive oxides have been shown to promote the formation H_2_ and CO in the final gas products of sucrose pyrolysis, which can further enhance the reduction of cathode active materials.^41–43^ The influence of sucrose content, temperature and heating duration of the roasting process and Li extraction is thoroughly studied. Furthermore, the impact of F and Al in the cathode active material is investigated to assess their influence on the roasting process, determining if additional pretreatments are required to achieve higher lithium recovery rates.

## Experimental

### Used LIBs and extraction of cathode stock material

The LIBs utilized in this study were obtained from a local electronics repair shop, specifically sourcing end-of-life iPhone batteries. The batteries were discharged in a 5 wt% NaCl (KATI coarse salt, 99.0%) solution for 24 hours to ensure complete discharging and lower the risk of short-circuiting and thermal runaway during dismantling. The discharged LIBs were manually dismantled and separated into cathode, anode, separator, and casing materials. The obtained cathode layers were crushed and sieved into two fractions: underflow (<250 µm) – rich with cathode active material, used for roasting experiments, and overflow (>250 µm) – comprising mainly of the aluminium current collector, not used in this study. The first stock of cathode active material was prepared and used to investigate the effects of sucrose content, roasting temperature, and heating time on the reduction of LCO and efficiency of Li recovery through water leaching. A simplified schematic of the whole process can be seen in Fig. S1 in the SI.

To study the possible effect of F and Al on the reduction process, a second stock batch of cathode active material was also prepared using the same procedure as described above. However, two additional pre-treatment steps were applied to the second batch of cathode active material to first remove the binder and then Al. Binder removal was carried out by dissolving the material in *N*-methyl-2-pyrrolidone (NMP, VWR, ≥99%) at 110 °C for 4 h, using a solid-to-liquid (S/L) ratio of 100 g L^−1^. Following binder removal, aluminium was selectively leached out with a 4 M NaOH solution (Sigma-Aldrich, ≥98%) at 60 °C for 4 hours, S/L 100 g L^−1^. After each treatment the materials were thoroughly washed with ethanol (Berner, ≥99.5%) and ultra-pure water to eliminate any residual solvent left behind after filtration.

### Roasting and water leaching of cathode active material

The cathode active material was mixed with sucrose (Dan Sukker, granulated beet sugar, Lithuania) at different dosages – 0, 5, 10, 15, and 20 wt% – in a planetary ball mill (Fritch Pulverisette, Germany) at 250 rpm, 3 × 5 min, with 5 min brakes in between to avoid the melting of sugar and to ensure a homogeneous mixture of active material and sucrose. The mixtures were roasted at temperatures between 250–600 °C under Ar flow, with a heating rate of 20 °C min^−1^ in a tube furnace (Nabertherm GmbH, Germany). The duration of the roasting process was varied between 0 to 120 min at target temperature. To obtain a more precise control over the roasting period, the tube furnace with built-in cooling ventilators was used, and the furnace heating area cover was opened as soon as the set roasting period was completed to ensure faster cooling. The roasted samples were then leached in ultra-pure water (60 min, 30 g L^−1^, 200 rpm) at room temperature. The Li leaching efficiency was determined with the following equation:11
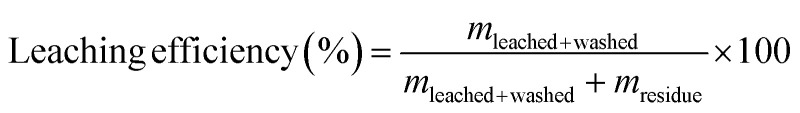
where *m*_leached+washed_ is the amount of Li in the water leaching and washing solution and *m*_residue_ is the residual Li left in the leach residue. To determine the Li concentration in the leach residue, dissolution in aqua regia (conc. HNO_3_ + conc. HCl with 1 : 4 ratio, S/L 100 g L^−1^, duration 24h, heated at 100 °C for the first 30 minutes, 100 rpm) was performed.

### Materials characterization

Phase composition analysis of the samples was done using X-ray diffraction (XRD, PANalytical; X'Pert3 Power, the Netherlands), with Cu K1 radiation, with an operation voltage of 45 kV and current 40 mA, scan angle of (2*Θ*) 5–90°, step size 0.026°, and a scan speed of 0.044° s^−1^, and analysed using HighScore Plus software. Precise chemical compositions of the samples were determined using flame atomic absorption spectroscopy (AAS) (Varian Spectra AA 220 FS) and Spectra AA software. Elemental analysis of samples was performed using a scanning electron microscope (SEM; Zeiss MERLIN, Austria) equipped with an energy-dispersive X-ray spectroscopy (EDX) detector (EDX-Flash 3001), operated at an acceleration voltage of 15 kV. TGA (Mettler Toledo TGA 1 STAR System, US) was performed on the sucrose in the temperature range of 25–800 °C, heating rate 10 °C min^−1^, and under N_2_ flow (20 mL min^−1^).

## Results and discussion

### Composition of cathode stock materials

**Fig. 1:**
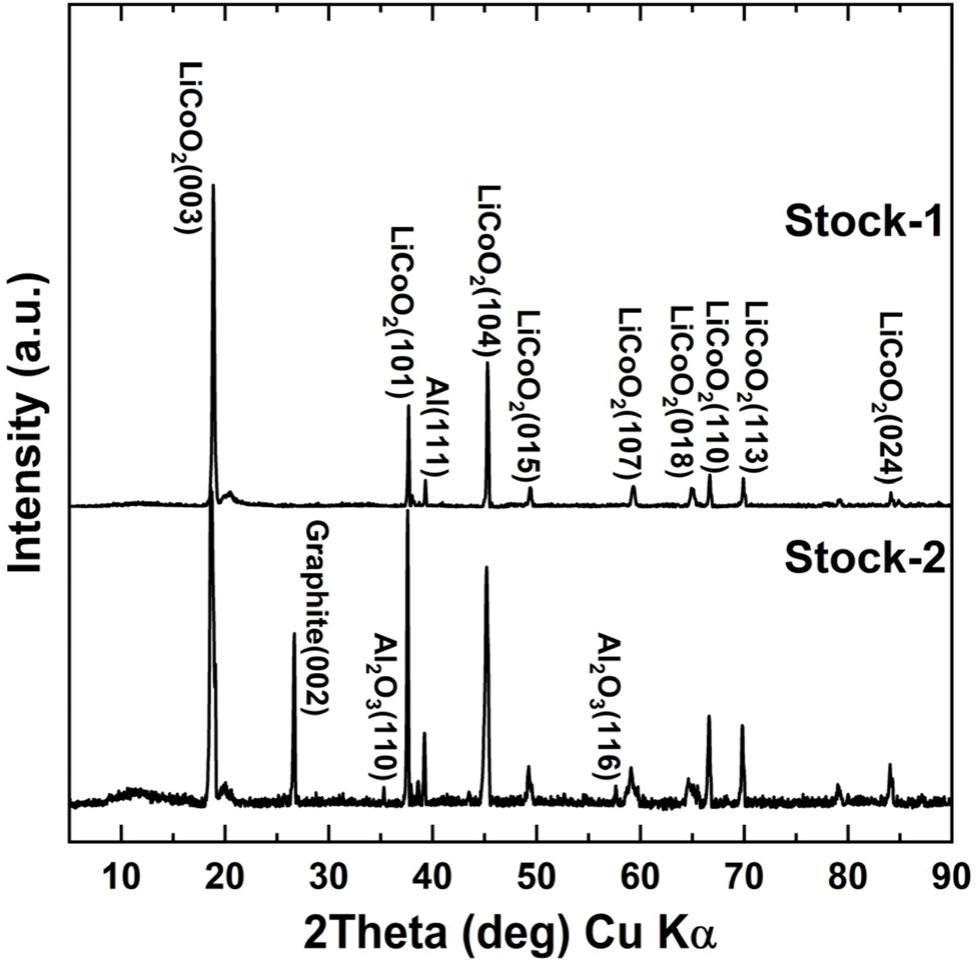
XRD diffractograms for Stock-1 and Stock-2.

Two stocks of cathode active material were prepared and used in this study: Stock-1 for optimizing roasting parameters and Stock-2 to study how the removal of F and Al during the pre-treatment of cathode active material affects the roasting process and lithium recovery efficiency. SEM-EDX analysis (Fig. S2 in SI) showed that the stock cathode active material contained Co, and O, along with Al, C, P, and F as the main additives. Li was not detected from the SEM-EDX analysis as only elements with an atomic number higher than B (*Z* = 5) can be detected at concentrations 0.1% or higher.^44^ Al originates from the Al current collector or from Al_2_O_3_ based additives or coatings, which are often used to improve thermal or structural stability of the cathode layer.^45^ C originating from the conductive carbon and binder, F from binder and electrolyte salt. For more details the Stock-2 SEM-EDX results with mapping images can be seen in the SI file (Fig. S2). XRD analysis of the stock materials (Fig. 1) confirmed the presence of LCO as the main crystalline phase in both samples. Minor diffraction peaks corresponding to carbon black or graphite were also observed in Stock-2, originating from the conductive carbon additives incorporated into the cathode formulation to enhance electrode conductivity.^46^ XRD analysis revealed that Stock-2 contained both Al and Al_2_O_3_, indicating the presence of an alumina surface coating (Al_2_O_3_) on the cathode material or oxidation of the Al foil.^47^ In contrast, Stock-1 exhibited signals corresponding mainly to metallic Al, likely originating from the Al current collector.

To gain a more comprehensive understanding of the chemical and crystalline composition of potential additives in the cathode active material, leaching with aqua regia was performed to dissolve the cathode active material. Both stocks exhibited a noticeable amount of undissolved residue after aqua regia treatment, with Stock-2 showing a significantly higher undissolved solid fraction (11.6 wt%) compared to Stock-1 (3.26 wt%), consistent with the earlier conclusion that Stock-2 contains carbon black or graphite, and also Al_2_O_3_, which are not soluble in aqua regia. SEM-EDX results in Fig. S3 shows that the aqua regia leach residue for Stock-1 consisted mainly of C (58.07 wt%) and F (22.97 wt%), originating from the conductive carbon and binder material polyvinylidene fluoride (PVDF).^48^ XRD results for both stocks aqua regia leach residues are presented in Fig. S4. In addition to the conductive carbons, also LiF, Al_2_O_3_, SiO_2_, boehmite (AlOOH), Co_3_O_4_ and small amounts of LCO were detected in the aqua regia leach residues of both stocks. The only notable difference was that Stock-2 contained significantly more crystalline carbon (graphite). The presence of Co_3_O_4_ and LiCoO_2_ in the aqua regia leaching residues may be attributed to the fact that, without dissolution or pyrolysis on the cathode material to prior remove the PVDF binder, the cathode active materials may remain coated with binder, which can prevent complete dissolution of metals.^49^ LiF is formed when LiPF_6_ in the electrolyte decomposes into LiF and HF that can further react with Li in the cathode to form more LiF.^50^ Boehmite, SiO_2_ and Al_2_O_3_ are often used in batteries as additives to cover the separator and active electrode materials, respectively, to enhance the Li-ion kinetics.^51–53^

### Roasting temperature effect on the lithium leaching efficiency

**Fig. 2:**
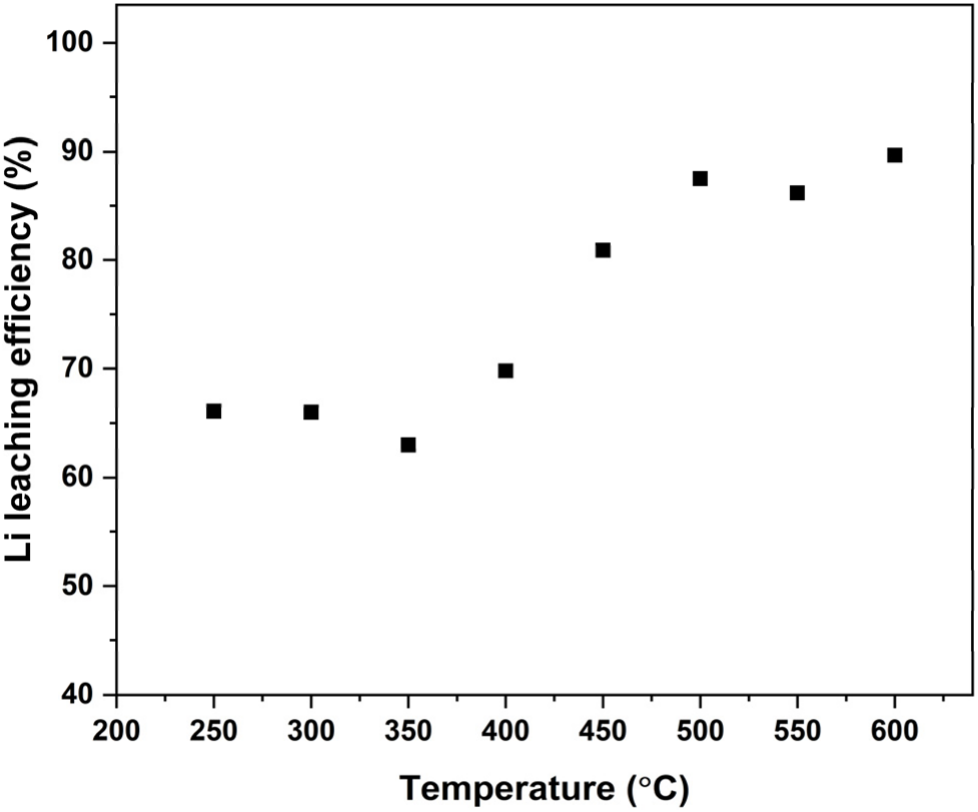
Li leaching efficiency *vs.* roasting temperature with 10 wt% sucrose.

The roasting temperature effect on the Li leaching efficiency was studied between temperatures 250 to 600 °C with a 10 wt% sucrose dosage and 60 min roasting time. From Fig. 2, it can be seen that relatively high Li leaching efficiency (66.1%) was already achieved at 250 °C. Interestingly, the Li leaching efficiency remained stagnant between 250 and 350 °C and started to slightly increase (69.8%) at 400 °C. A noticeable increase in the Li leaching efficiency was observed when the roasting was performed at temperatures over 400 °C, achieving efficiencies of 80.9% at 450 °C and 87.5% at 500 °C, respectively. Increasing the roasting temperature even further, from 500 °C to 600 °C, resulted in a slight increase to 89.7% in the Li leaching efficiency. Previously, it has been reported that carbothermic and hydrogen reduction of LCO (reactions 1–10, presented in the introduction section) at low temperatures, up to 400 °C, have resulted in low Li recovery rates, mostly under 30%.^25–30^ In our experiments the relatively high LCO reduction, already at low temperatures, most likely occurs through the intermediate products of sucrose decomposition. Sucrose itself starts to dehydrate and caramelize at around 200 °C and reaches its decomposition point at around 250 °C, where the mass loss is the fastest based on TGA measurements (Fig. S5). Starting from 300 °C, mass change slows down and the decomposition reaction speed decreases.^40^ A similar effect was observed by Yunze Zhao *et al.*, who used macadamia nut biomass to roast NMC111.^54^ In their study, pyrolysis of the biomass began at 190 °C, generating reducing agents that partially reduced the NMC111 cathode active material as early as 300 °C. The Li leaching efficiency starts to increase again from 400 °C, due to released gaseous products (mostly CO and H_2_) and carbon species becoming more reductive at higher temperatures. For comparison, Ji X. *et al.*^35^ observed similar behaviour when roasting with glucose at 450–500 °C, however, an even higher Li leaching efficiency, up to 98.3%, was achieved at 550 °C. The very high lithium leaching efficiency achieved by Ji X. *et al.* is likely due to additional pretreatment steps before roasting such as dissolution of PVDF in *N*-methyl-2-pyrrolidone and subsequential calcination at 700 °C to remove graphite and other organic compounds.^55^ This resulted in the cathode stock having less additives that react with Li and form insoluble Li compounds during the roasting process and thus affect the Li leaching efficiency.

**Fig. 3:**
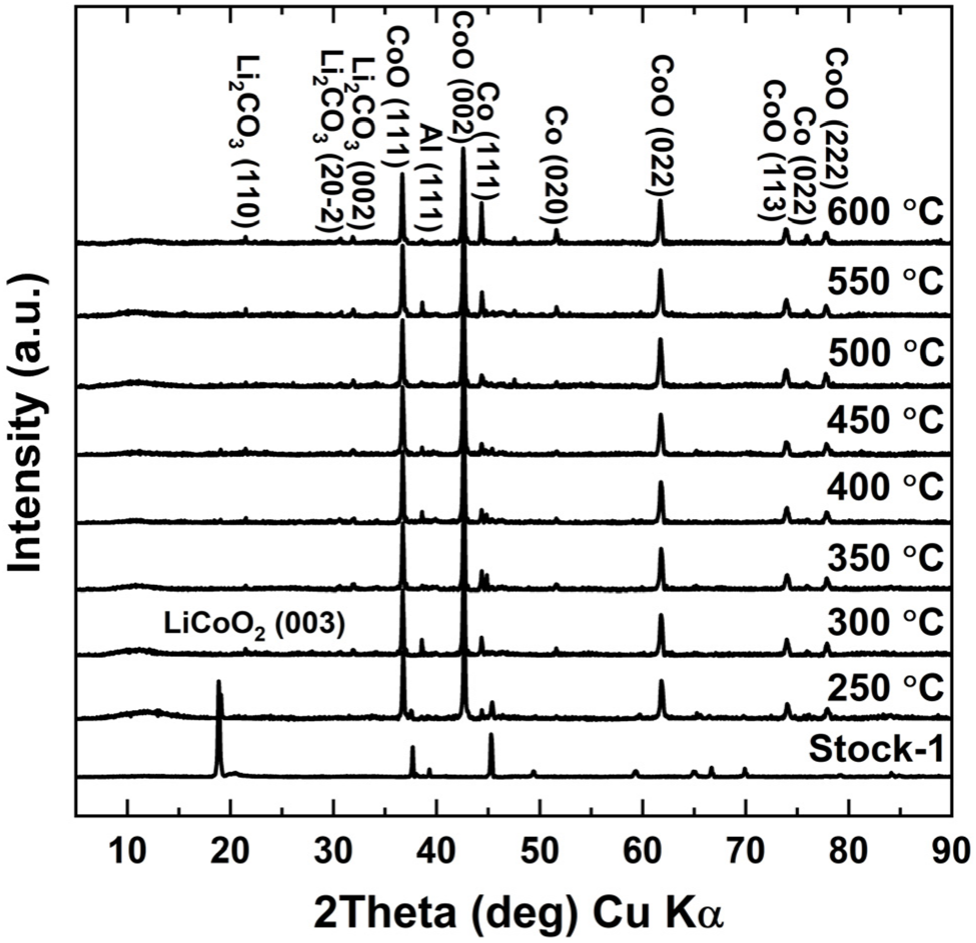
XRD diffractograms of roasted samples – roasted at different temperatures with 10 wt% sucrose dosage and 60 minutes roasting time.

**Fig. 4:**
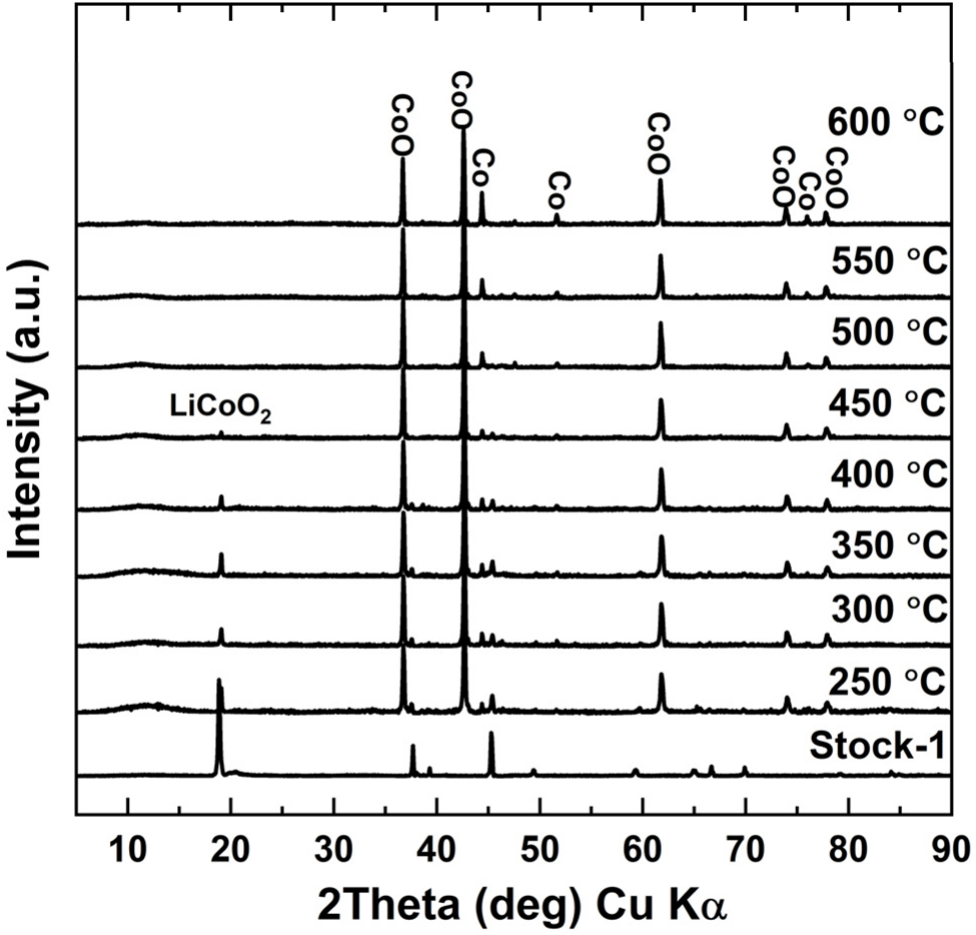
XRD diffractograms of water leaching residue samples – roasted at different temperatures with 10 wt% sucrose dosage and 60 minutes roasting time.

When analysing the XRD diffractograms of roasted samples in Fig. 3, it was found that, besides the sample roasted at 250 °C, none of the samples indicated any visible LCO peaks. However, when looking at the XRD diffractograms for the water leaching residues in Fig. 4, LCO peaks appeared again for all the samples roasted below 500 °C. The LCO peak intensities in the leaching residues correlated well with the calculated Li leaching efficiencies, with LCO peaks decreasing on the XRD diffractograms as the Li leaching efficiency increased. No LCO peaks were visible when roasting was performed at 500 °C or higher, showing that the cathode active material was completely reduced into Li_2_CO_3_, CoO, and Co, which are consistent with the typical peaks seen after roasting LCO with organic reductants.^33–35^ This indicated that the XRD diffractograms for water leaching residues acted as a good indicator on the roasting efficiency of LCO. From the XRD analysis it was also concluded, that with even further increase in the roasting temperature, metallic Co peaks became more pronounced and visible in both the roasted and water leached samples, most distinguishable at 600 °C. This indicated, that at higher temperatures, Co oxides were further reduced to their metallic forms.

To further understand why the Li leaching efficiency plateaued at around 90%, the aqua regia residues of the roasted and water leached samples were studied more closely. Analysis of the Stock-1 aqua regia residues revealed the presence of both Al_2_O_3_ and SiO_2_, which can react with Li to form various insoluble compounds, such as LiAlO_2_,^56^ Li_4_SiO_4_,^57^*etc.* XRD diffractograms of the aqua regia residues (Fig. S6) indicate that α-LiAlO_2_ had started to form at 450 °C. At lower temperatures, residual LCO peaks, which is similar in structure to α-LiAlO_2_, were present as was seen in the stock aqua regia residues. As the temperature increased, the intensity of α-LiAlO_2_ peaks also increased, suggesting progressive formation of this phase. Starting from 500 °C, a second amorphous phase of γ-LiAlO_2_ was also detected, becoming more prominent as temperature increased to 600 °C. This suggests that although higher temperatures can facilitate the reduction of the cathode active material, they will also promote the formation of insoluble Li–Al complexes, which can negatively affect the Li recovery. The SEM-EDX mappings in Fig. S7 to S12 showed that Al and Si concentrations in the aqua regia residue were significantly higher for the sample roasted at 500 °C compared to 350 °C, although the overall amount of the residue had degreased. This difference comes most likely from the decomposition of PVDF. This was also supported by the 350 °C sample having a similar amount of Co (1.20 wt%) compared to the stock aqua regia residue while the Co composition at 500 °C had reduced by half (0.58 wt%). The amount of F present in both samples reduced significantly for both samples, indicating the formation of LiF, which was leached out during water leaching and aqua regia. XRD results of the aqua regia residues in Fig. S6 also confirmed this, as LCO was still seen to be present in the aqua regia residues most likely due to PVDF coating the cathode active material as was seen in the stock aqua regia residues. When comparing the total amount of Li in the water leaching and aqua regia solution to the starting cathode mass, no trend was seen in the Li concentration *vs.* temperature, as the Li composition jumped randomly between 4.0–4.4%. This difference of around 10% can come from the heterogeneous nature of the stock which makes it hard to estimate the total loss of Li into the insoluble complexes. However, the total amount of Li measured in the experiments fell in the range that was measured for Stock-1 (4.0–4.8%). The aqua regia residue was also releached in aqua regia again to check possible Li traces, but the measured Li concentration was below the detection limit.

### Effect of sucrose dosage on lithium leaching efficiency

**Fig. 5:**
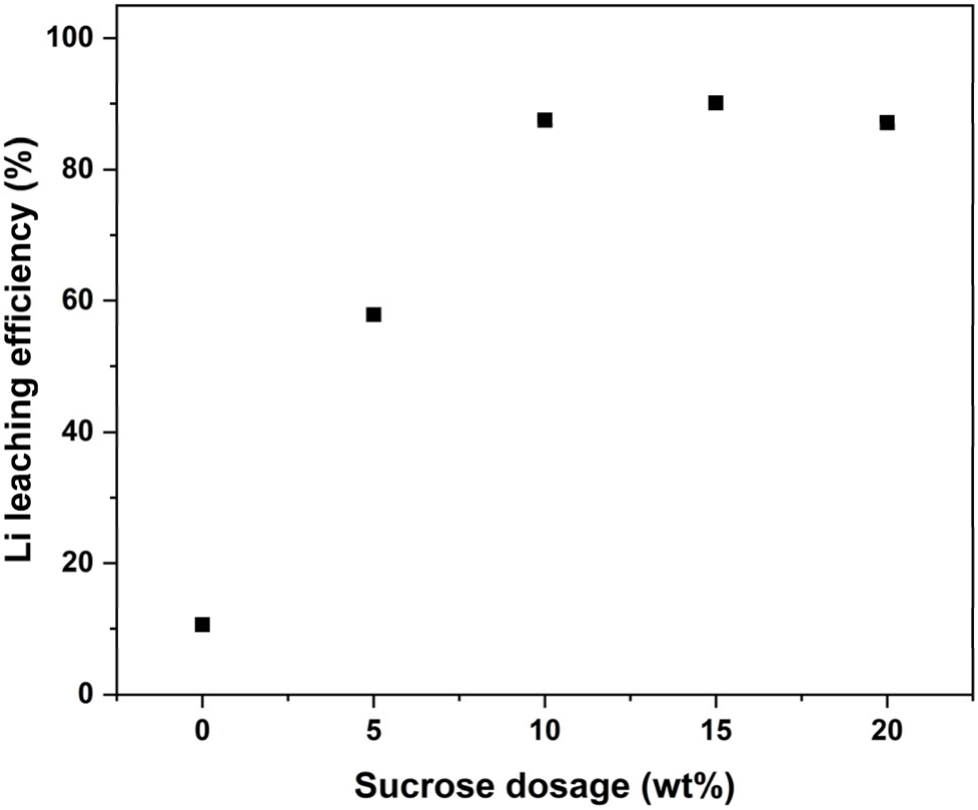
Li leaching efficiency *vs.* sucrose dosage at 500 °C and 60 min roasting time.

**Fig. 6:**
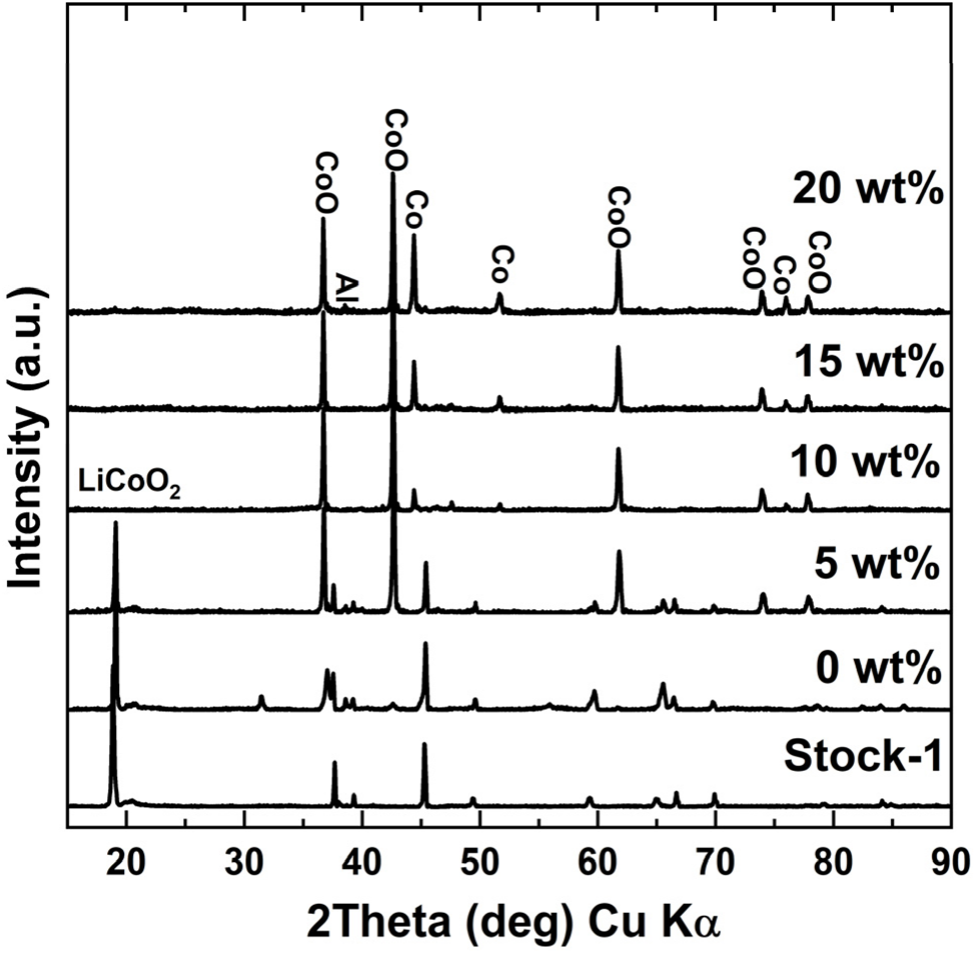
XRD diffractograms of water leaching residue samples – roasted at different sucrose dosages at 500 °C and 60 minutes roasting time.

Based on the roasting temperature optimization in previous section, it was found that in the case of 10 wt% sucrose dosage, 500 °C was the optimal roasting temperature, which resulted in a Li leaching efficiency of 87.5%. To better understand the role of sucrose during the roasting experiments, the effect of sucrose dosage was also studied by varying it from 0 to 20 wt%. Without any added sucrose, a Li leaching efficiency of 10.6% was achieved after the roasting process. This shows that the organics, carbon, and possibly Al content in the cathode stock can somewhat reduce the cathode, however, the effect is very low and additional reductant is required to achieve efficient Li recovery. From Fig. 5, it can be seen that Li leaching efficiency enhanced noticeably from 10.6% to 87.5% with increasing the reductant content to 10 wt%, after which only a slight increase to 90.1% was seen with 15 wt% of sucrose in the mixture. With even further increase in sucrose dosage, Li leaching efficiency decreased to 87.1%. This occurred because sucrose started to foam with higher dosages during roasting, causing the mixture's volume to expand. As a result, some of the cathode material did not reduce effectively due to insufficient contact with the reductant. The foaming effect was the reason why dosages higher than 20 wt% could not be tested, as the mixture foamed over the sample holder and material was lost in the tube and gas line. When looking at the XRD diffractograms for water leach residues in Fig. 6, small peaks for LCO were noticed with 20% sucrose dosage, confirming that some of the cathode had not been reduced. Additionally, higher peak intensities for metallic Co were seen when sucrose dosage increased, showing further reduction of CoO into metallic Co, when more reductant was used. For a safety concerns, this should be avoided as metallic Co can produce H_2_ in following leaching steps.^17^

### Effect of roasting time on lithium leaching efficiency

**Fig. 7:**
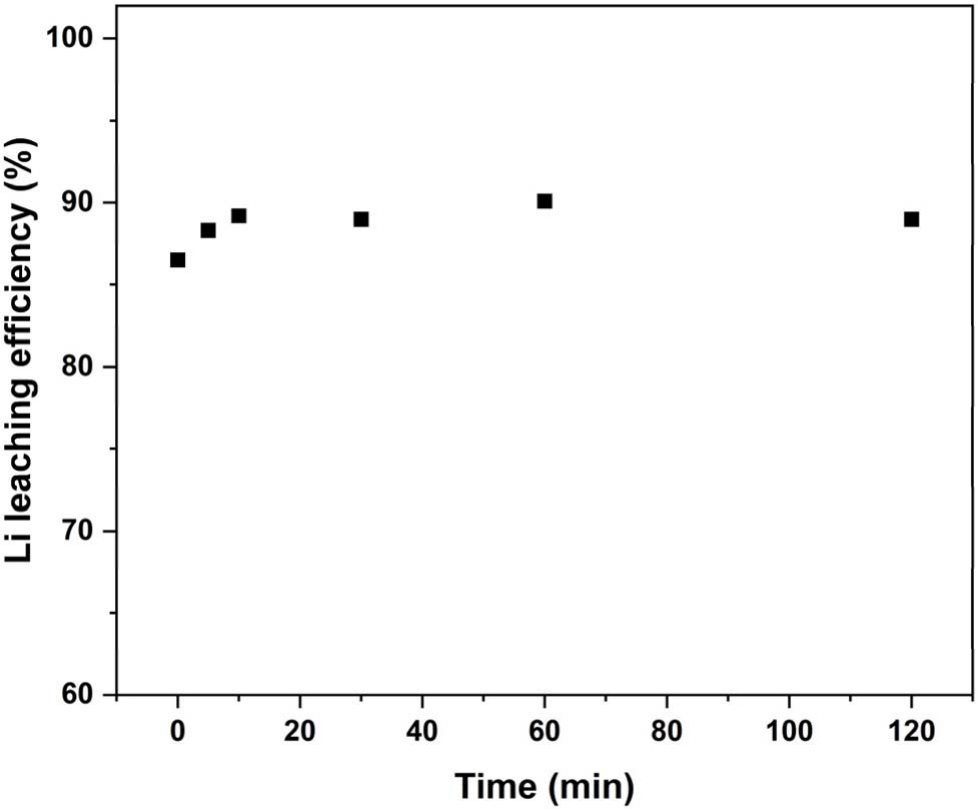
Li leaching efficiencies at heating times from 0 to 120 min at 500 °C and 15 wt% sucrose.

**Fig. 8:**
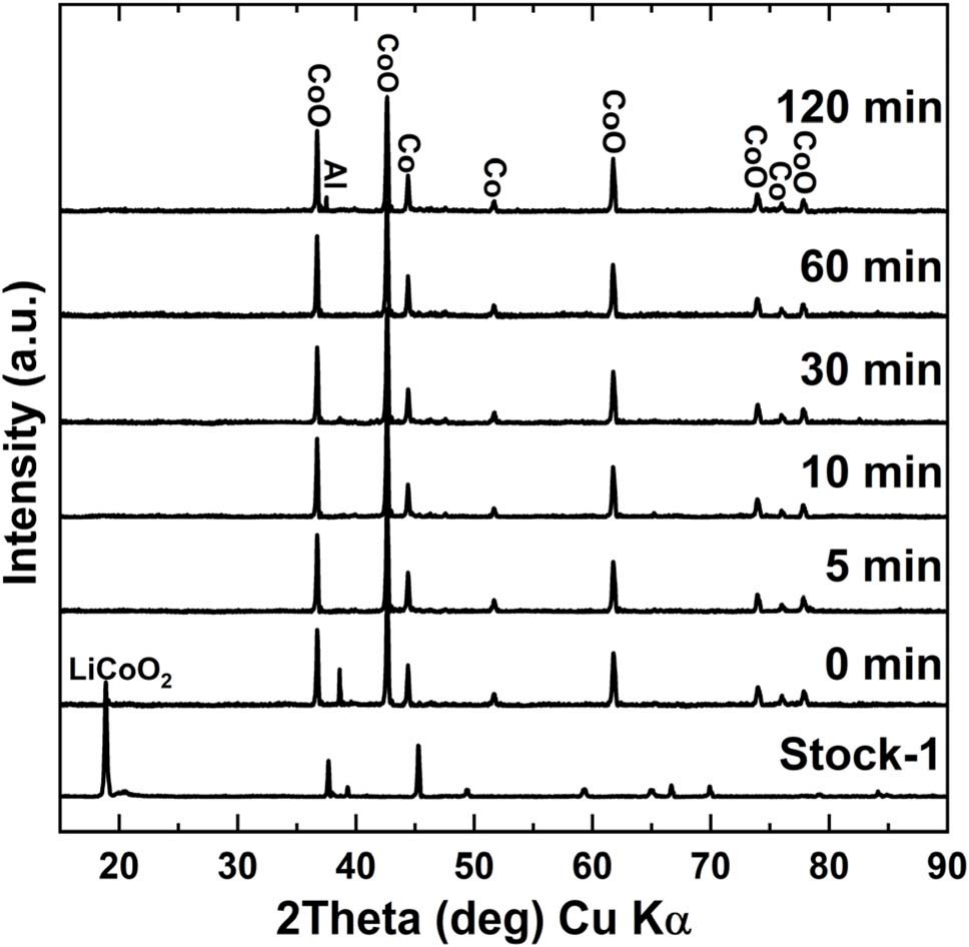
Water leaching residues of samples roasted from 0 to 120 min at 500 °C with 15 wt% sucrose dosage.

The third roasting condition that can potentially influence the process and Li leaching efficiency is the duration of roasting. Based on previous roasting optimisation experiments, a temperature of 500 °C and 15 wt% sucrose dosage was chosen, and the roasting time was varied between 0 to 120 min. Very interesting results were obtained as can be seen in Fig. 7, with the lowest Li leaching efficiency achieved being 86.5% when the tube furnace was immediately opened for cooling after reaching the target roasting temperature. The temperature profile for the roasting experiment with just heating up to 500 °C and no holding time can be seen in Fig. S13. From there it is seen that the temperature was above 400 °C for approximately 7.5 minutes and over 450 °C for just under four minutes. An increase of only 3.6% was seen in Li leaching efficiency when the roasting duration was increased to 60 minutes. When looking at the masses of the roasted products, no significant changes were seen between different holding times. This suggests that both the sucrose decomposition and LCO reduction reactions occur very rapidly during roasting, thus not enhancing the Li leaching efficiency noticeably with further reduction as heating duration increases. The slight increase in Li leaching efficiency (from 86.5% to 90.1%) can be explained by further reduction taking place due to carbon from the pyrolysis of sucrose and the decomposition of the binder. XRD results for water leaching residues in Fig. 8 also showed that no LCO could be detected after just heating up the roasting mixture to 500 °C. Co peaks intensified as roasting time increased as well, indicating that longer roasting times only help to further reduce CoO. In addition to that, the aqua regia residue diffractograms seen in Fig. S14, also showed that at 120 minutes roasting time, γ-LiAlO_2_ started to become more noticeable showing that longer holding times can result in Li reacting more with the impurities in the roasting mixture and forming unwanted compounds. The peak intensities for α-LiAlO_2_ were also found to be the lowest at the shortest holding times, increasing slightly as the holding time was increased. Such short roasting times have not been reported in the literature previously. The optimal roasting conditions for different organic reductant roasting systems for LCO found in literature can be seen in Table S1 There it can be seen that the average best results for different roasting systems is achieved with holding times of 1–2 hours at similar temperatures.^17,26–30,35^ The large reduction in roasting time can significantly decrease energy consumption and increase the overall capacity for the process, resulting in significant reductions in operational costs.

### Effect of impurities on lithium leaching efficiency

Even though high Li leaching efficiencies were achieved during these experiments, about 10% of the Li still remained in the residue after water leaching due to the formation of LiAlO_2_ and LiF. The solid to liquid ratio (S/L) for the water leaching experiments was chosen so that all of the Li should completely dissolve if fully converted into the form of Li_2_CO_3_ (solubility 13.3 g L^−1^ at 25 °C (ref. 58)) during the roasting, meaning that the remaining Li in the filter cake had to be in an insoluble form in water. It has been previously shown that with a lower carbon content and higher Al concentrations, LiAlO_2_ can form during the roasting process at temperatures 550–750 °C.^26^ The formed LiAlO_2_ is in an amorphous state and cannot be detected easily in XRD or dissolved in water. LiF is typically formed from the decomposition of the electrolyte salt LiPF_6_ and the PVDF binder, which release HF that can potentially react with lithium to form LiF,^25^ which has very low solubility in water (at 25 °C, 1.6 g L^−1^ for LiF) and would thus lower the Li leaching efficiency.^58^ This can affect the Li leaching efficiency calculation used in this study slightly (eqn (11), presented in experimental section) by slightly increasing the calculated Li leaching efficiency, as some of the undissolved Li still remains in the aqua regia residue. However, this loss was estimated to be below 1% and calculating the Li leaching efficiency based on the overall Li composition of the stock would be more inaccurate, as the stock material was found to be heterogeneous and the Li percentage varying from 4.0% to 4.8%. To further test the hypothesis that the remaining undissolved Li was possibly caused by LiF and LiAlO_2_, a new batch of cathode, named as Stock-2 was made using the same procedure as before, but additionally two different pre-treatment steps were performed before roasting experiments: (1) dissolution of PVDF in NMP, and (2) NMP treated cathode leached in NaOH. The roasting conditions chosen for the tests were 500 °C roasting temperature, 15 wt% sucrose dosage and 60 minutes roasting time. The longer roasting time was chosen to avoid any uncertainties that shorter roasting times could cause and to have more comparable results with the first stock, as most of the previous systematic experiments had been done with a roasting time of 60 minutes.

**Fig. 9:**
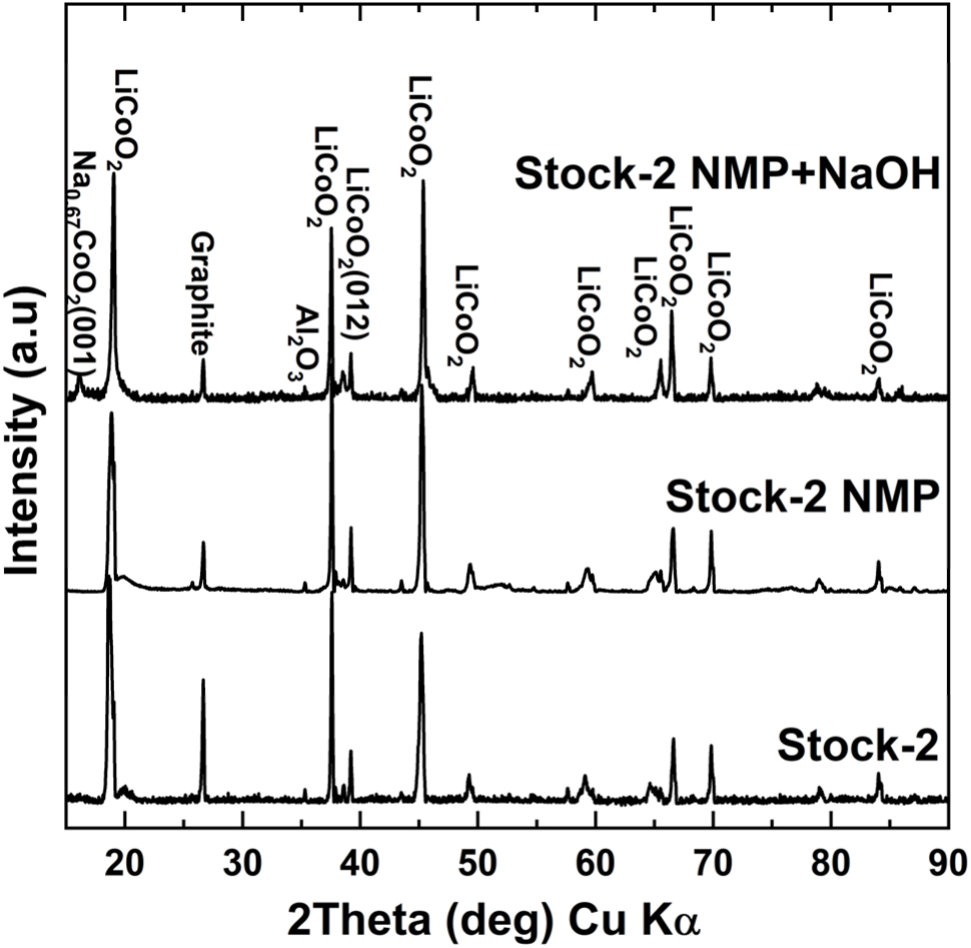
XRD diffractograms of Stock-2 after NMP and NaOH dissolution to remove F in the binder and Al.

The new cathode batch (Stock-2), originated from different iPhone batteries than Stock-1 and, therefore, had a slightly different composition as some graphite and Al_2_O_3_ were detected (Fig. 1) in the stock. The new batch was also tested for other elements (Ti, Zn, Mn, Ni, Fe, Cu) using both XRF and flame AAS, but all concentrations for these elements remained below 10 ppm, which was the detection limit. Although the composition for the Stock-2 was slightly different from Stock-1, Li leaching efficiency after roasting the untreated stocks at 500 °C, 15 wt% sucrose dosage, and 60 minutes roasting time remained in similar range – 90.1% for Stock-1 *vs.* 86.1% Stock-2, respectively. After dissolution in NMP, the Li leaching efficiency increased to 91.6%. SEM-EDX results (Fig. S15) showed that no F was detected in the Stock-2 after NMP dissolution, indicating the removal of PVDF was successful and the formation of LiF was avoided during roasting. Additionally, the P concentration had also considerably decreased (0.54% *vs.* 0.13%) indicating that most of the electrolyte salt, LiPF_6_, had also dissolved into the NMP solution. The Li leaching efficiency further increased to 94.5% after treating the cathode with NaOH. Additionally, XRD results in Fig. 9 showed new peaks in the cathode mix after the NaOH pre-treatment, which was found to be Na_0.6_CoO_2_. This is most likely caused by Na ions intercalating into vacancies in the LCO structure or by replacing Li ions due to the chemical potential from the high concentration of the NaOH solution. This effect has been used in ion-exchange synthesis for cathode materials to synthesize layered oxide structures for LIBs with better control and less defects compared to high-temperature sintering.^59^ Although this could imply, that some of the Li could had been leached out of the cathode during the NaOH treatment, no Li was detected in the leach solution and intercalation likely occurred due to Li vacancies in the LCO structure.

XRD results of water leaching residues in Fig. S17 showed almost no differences between the residues after different pre-treatments, except for small peaks for hexagonal Co becoming visible after PVDF had been removed. XRD diffractograms for aqua regia residues in Fig. S17 comprised mostly of graphite and Al_2_O_3_, with Al_2_O_3_ peaks reducing in intensity relative to graphite after each pre-treatment. Indicating that the NaOH treatment removed some of the Al, presumably Al originating from the current collector. However, the total removal of Al from the stock, was unsuccessful as active material contained also other Al species, for example boehmite (AlO(OH)). Boehmite is commonly used in lithium-ion batteries to coat separators and electrode sheets, improving both performance and safety. Boehmite remained after 4 M NaOH treatment, consistent with its slow alkaline dissolution,^60^ whereas heating above ∼500 °C dehydroxylates boehmite to transition alumina (mainly γ-Al_2_O_3_).^61^

When comparing the results in this work to other studies on roasting LCO with organic reductants, Li leaching efficiencies close to 90% or higher were all achieved, when the cathode active material had been removed from the Al foils through either NMP dissolution or pyrolysis, or the material used for the experiments was raw battery grade LCO purchased from a supplier. Separation before crushing can result in lower Al concentration in the remaining cathode active material due to the foils being manually removable. This is also supported by the fact that the lowest result (88.7%) achieved in Table S1 had crushed the cathode prior to dissolution in NMP. Although removal Al and F through additional pre-treatments can increase the overall Li recovery efficiency, the treatments themselves are highly chemical and energy intensive and can result in excessive costs and waste during recycling. In addition to that, LiAlO_2_ and LiF can already form during several pre-treatment processes prior to roasting, which can further reduce recovery efficiencies. These can include crushing of spent LIBs, electrolyte evaporation or heat treatments to remove PVDF or to maximise the separation efficiency for flotation, where either LiPF_6_ can start to decompose or temperatures reach high enough for Li to start reacting with Al.^62^ From this it can be concluded that to maximise Li recovery through roasting, removal of Al and F is necessary but new methods, either through dissolution or milder heat treatments, need to be developed to reduce the cost and environmental footprint of the pre-treatment process and to maximise Li recovery.

## Conclusions

The rapidly increasing demand for lithium-ion batteries raises serious concerns with material supply, appropriate disposal, and recycling of waste batteries. Current industrial solutions have mainly focused on recovering metals like cobalt, nickel, and copper while putting less emphasis on lithium recovery. In this study, the possible selective recovery of Li from LiCoO_2_ cathode active material through roasting with food grade sucrose was explored. The effect of roasting temperature, sucrose dosage, and roasting time on the Li leaching efficiency in water was studied. In addition to that, the effect of impurities such as Al and F on the recovery efficiency of Li were tested and analysed as well. The optimum conditions for the process were determined to be 500 °C for 60 minutes with 15 wt% sucrose dosage, achieving a Li leaching efficiency of 90.1%. Further increasing the roasting temperature showed no improvement on the Li leaching efficiency and resulted in the formation of more LiAlO_2_ which is insoluble in water. Roasting time optimisation showed that the roasting reaction occurs very rapidly with the lowest Li leaching efficiency being 86.5% when only heating the cathode mixture to the target temperature. The low dependency on roasting time significantly reduces energy requirements and throughput when compared to other roasting solutions and can make it a viable option for the industry to adapt. In addition to that, longer holding times again showed the increased formation of LiAlO_2_. Further improvement on the Li leaching efficiency was achieved when additional pre-treatment steps to remove F and Al were performed. The Li amount in the water leaching solution increased to 91.6% after NMP dissolution and further to 94.5% when leaching out Al in NaOH as well before roasting. Comparison with other works also found that to achieve Li leaching efficiencies over 90% using organic reductants, costly and environmentally intensive pre-treatments need to be done to remove the binder and Al foil. New methods need to be developed to remove these elements prior to heat treatment or reduce their reactivity with Li in order to maximise Li recovery from waste LIBs. Overall, using sucrose for roasting was found to be a cost-efficient and promising option for targeted Li recovery, providing relatively low roasting temperatures and very short roasting times.

## Author contributions

M. J.: conceptualization, investigation, and writing. K. L.: conceptualization, funding acquisition, supervision, investigation, and writing. V. M.: investigation.

## Conflicts of interest

The authors have no conflicts of interest to declare.

## Supplementary Material

RA-016-D5RA08726K-s001

## Data Availability

The data supporting this article have been included as part of the supplementary information (SI). Further data are available upon request from the authors. Supplementary information is available. See DOI: https://doi.org/10.1039/d5ra08726k.
