# Supplementary material for: Early-stage extraction of lithium from LCO cathodes via sucrose-assisted reductive roasting
Source: RSC Adv. 2026 Jan 2;16(1):872–82. doi: 10.1039/d5ra08726k (PMC12757914; doi:10.1039/d5ra08726k)
Supplement: RA-016-D5RA08726K-s001 [file RA-016-D5RA08726K-s001.pdf]

Supplementary Information (SI) for RSC Advances.
This journal is © The Royal Society of Chemistry 2025

Early-Stage Extraction of Lithium from LCO Cathodes via Sucrose-Assisted Reductive Roasting

Martin Jantson[1, 2], Kerli Liivand[1*], Valdek Mikli[2]

[1] National Institute of Chemical Physics and Biophysics, 12618 Tallinn, Estonia

[2] Department of Materials and Environmental Technology, Tallinn University of Technology, 19086 Tallinn, Estonia

Keywords: lithium-ion batteries; recycling; roasting; lithium recovery; lithium; lithium cobalt oxide (LCO); pyrometallurgy; green chemistry; metal reduction; circular economy.

***Corresponding authors:** kerli.liivand@kbfi.ee;

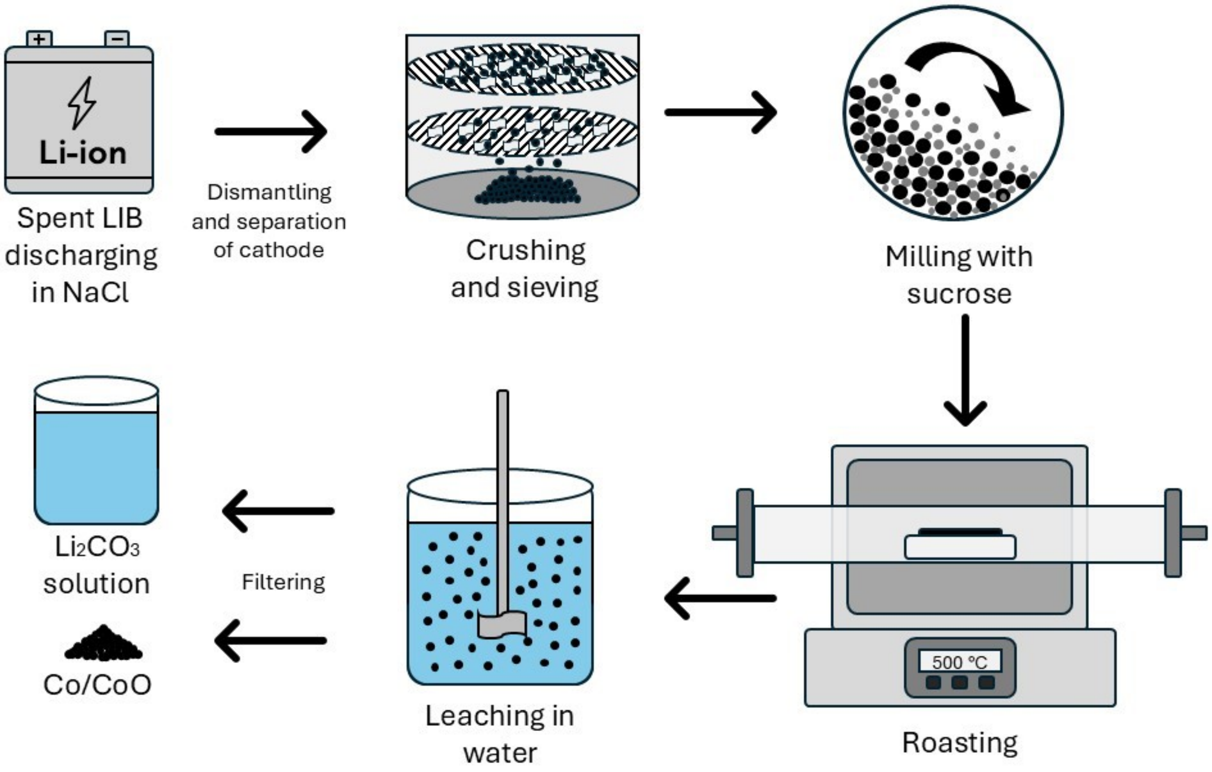

Figure S1. Graphical illustration of the pathway for targeted separation of lithium from LCO cathode by roasting with sucrose.

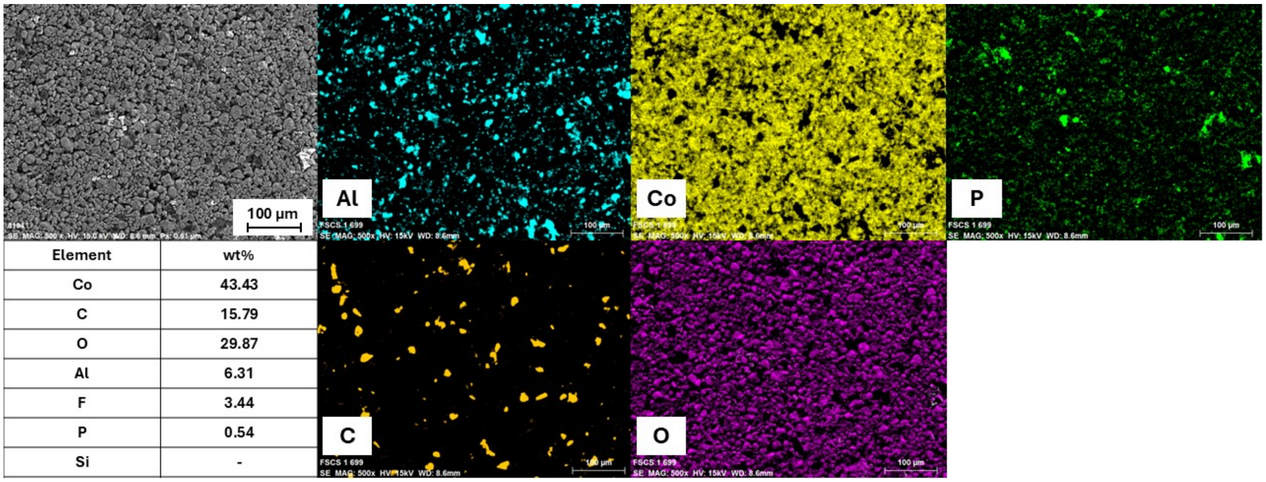

Element	wt%
Co	43.43
C	15.79
O	29.87
Al	6.31
F	3.44
P	0.54
Si	-

Figure S2. SEM-EDX micrographs and elemental composition for Stock-2.

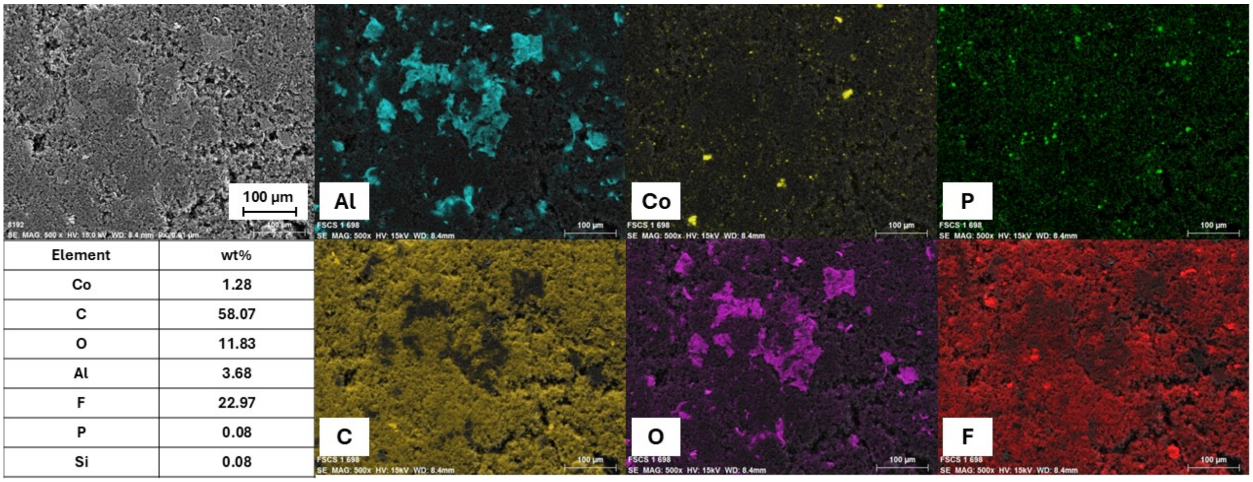

Element	wt%
Co	1.28
C	58.07
O	11.83
Al	3.68
F	22.97
P	0.08
Si	0.08

Figure S3. SEM-EDX micrographs and elemental composition for Stock-1 AR residue.

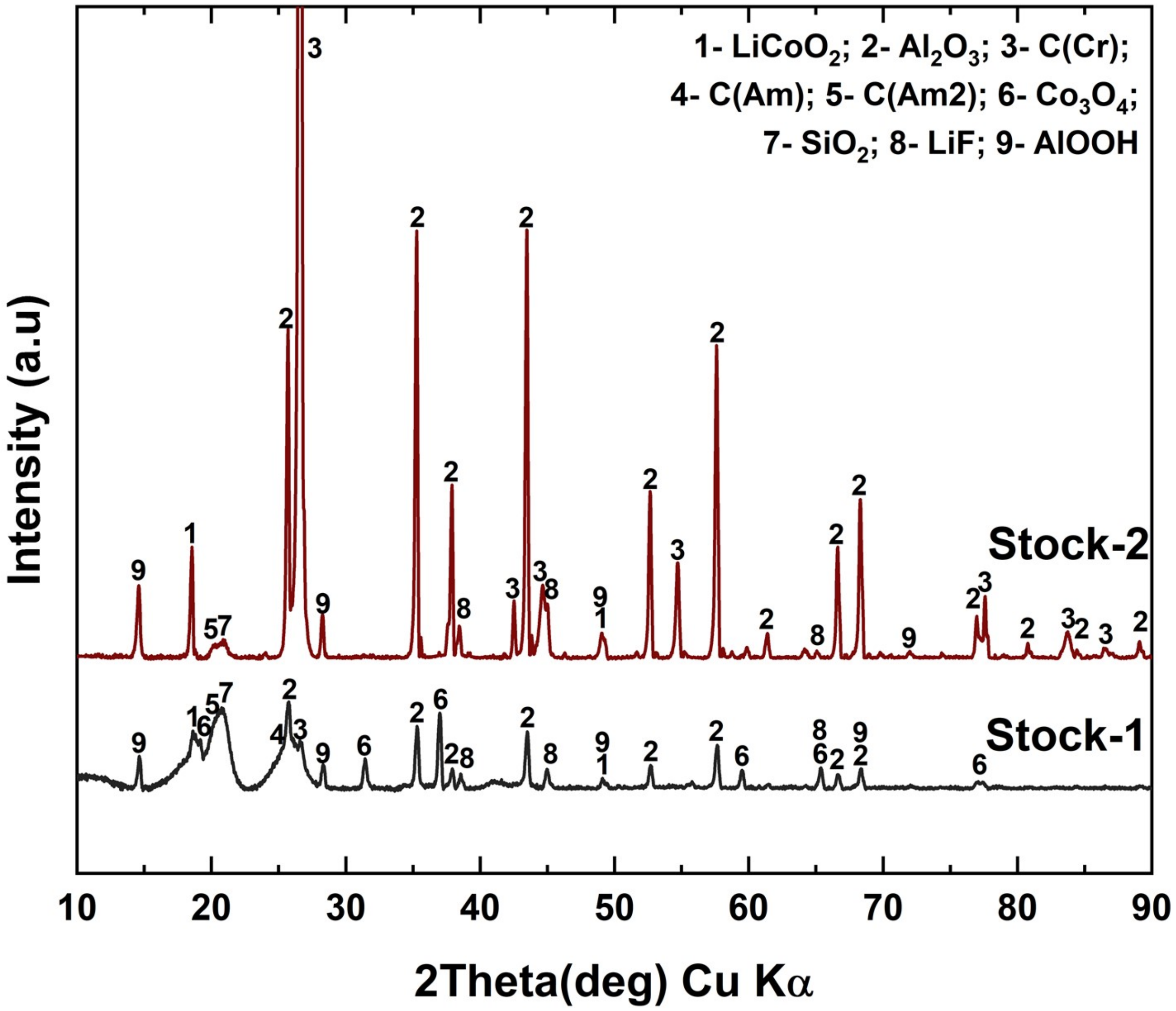

Figure S4. XRD diffractograms for both stock materials aqua regia leaching residues.

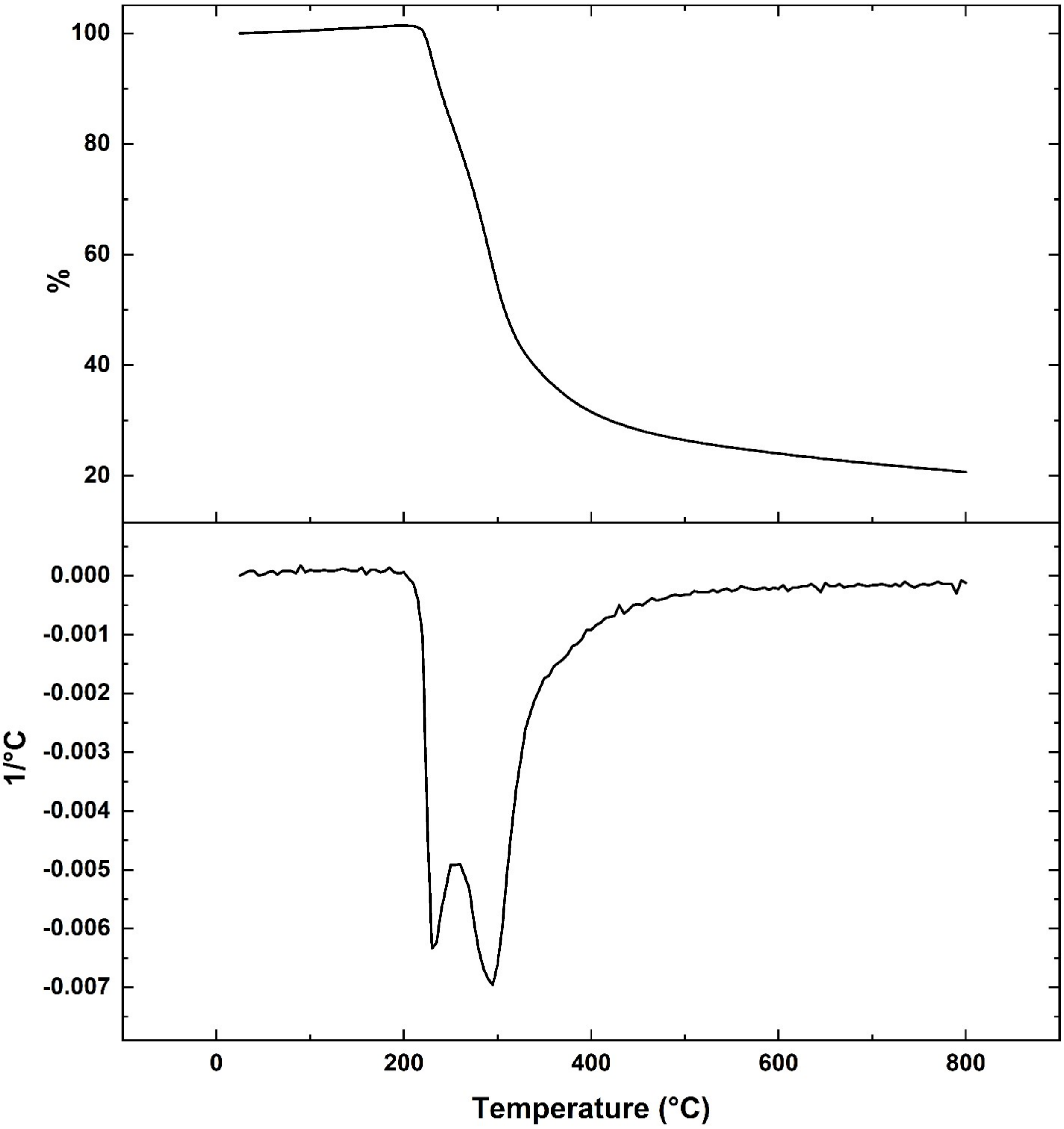

Figure S5. TGA profile for sucrose.

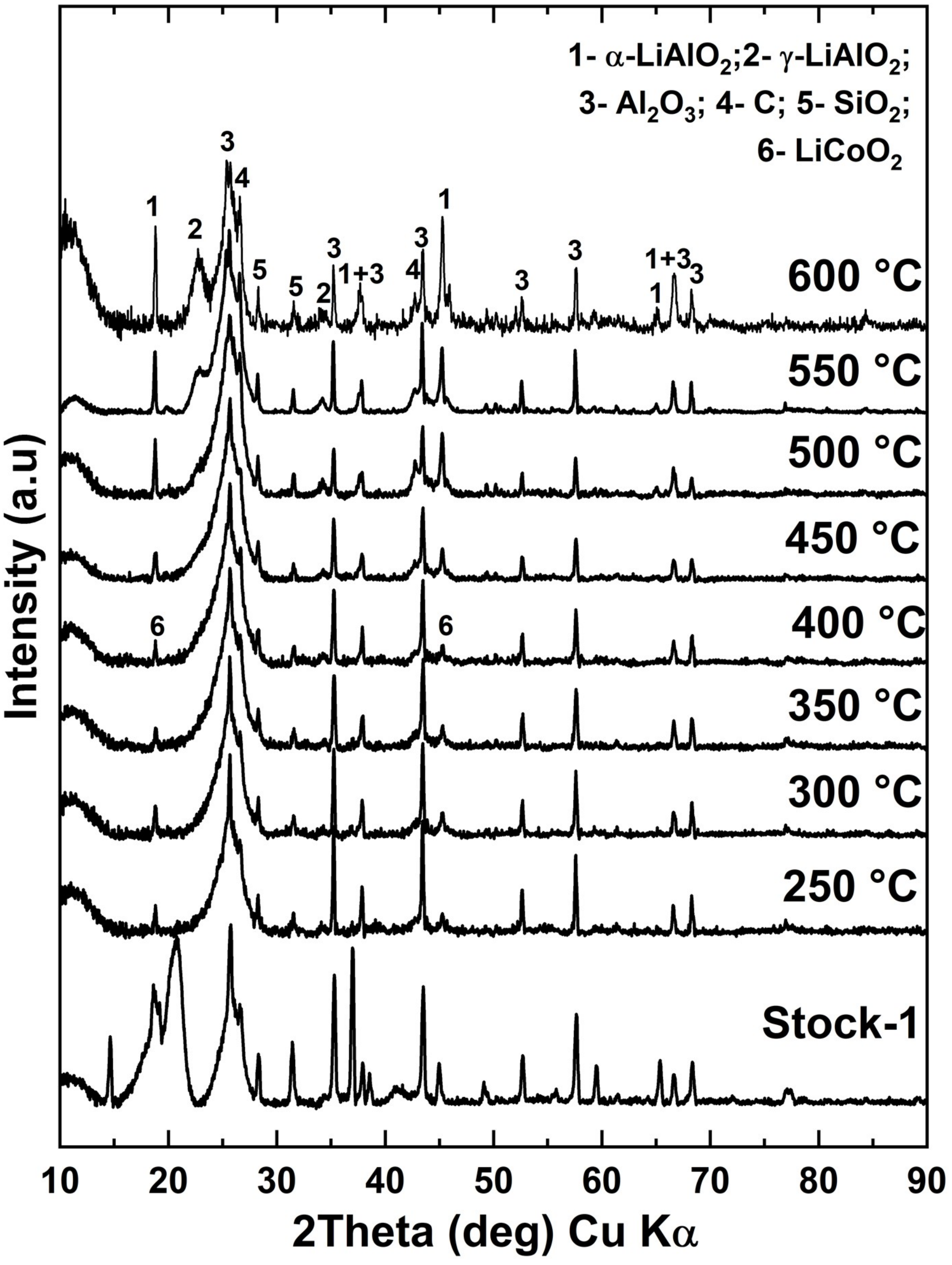

Figure S6. XRD diffractograms of aqua regia residues from temperature optimization experiments. All samples were roasted with 10 wt% sucrose for 60 minutes.

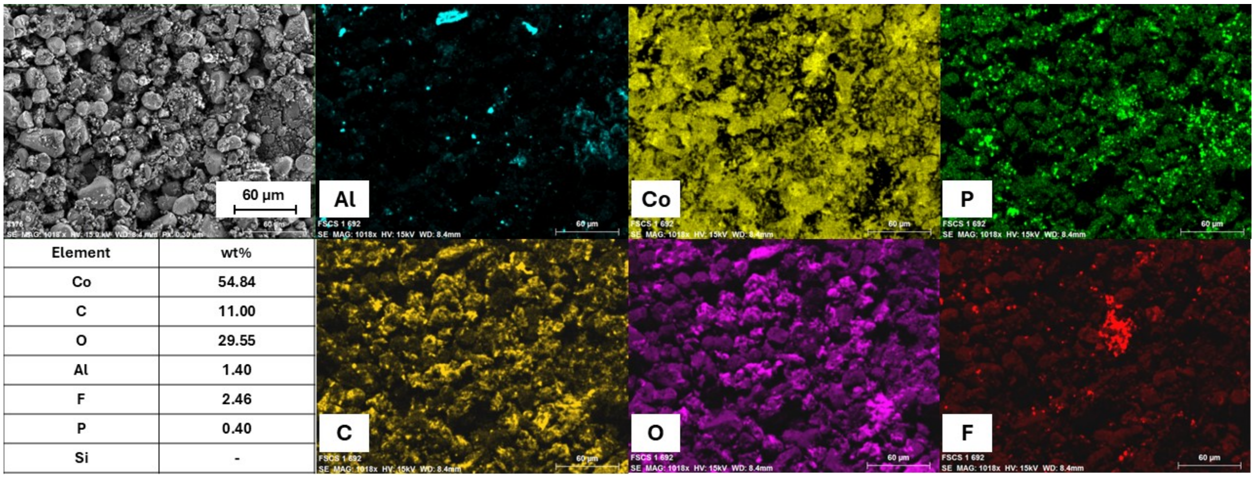

Element	wt%
Co	54.84
C	11.00
O	29.55
Al	1.40
F	2.46
P	0.40
Si	-

Figure S7. SEM-EDS micrographs and elemental composition for roasted cathode - roasted at 500 °C, 10 wt% sucrose dosage, 60 min roasting time.

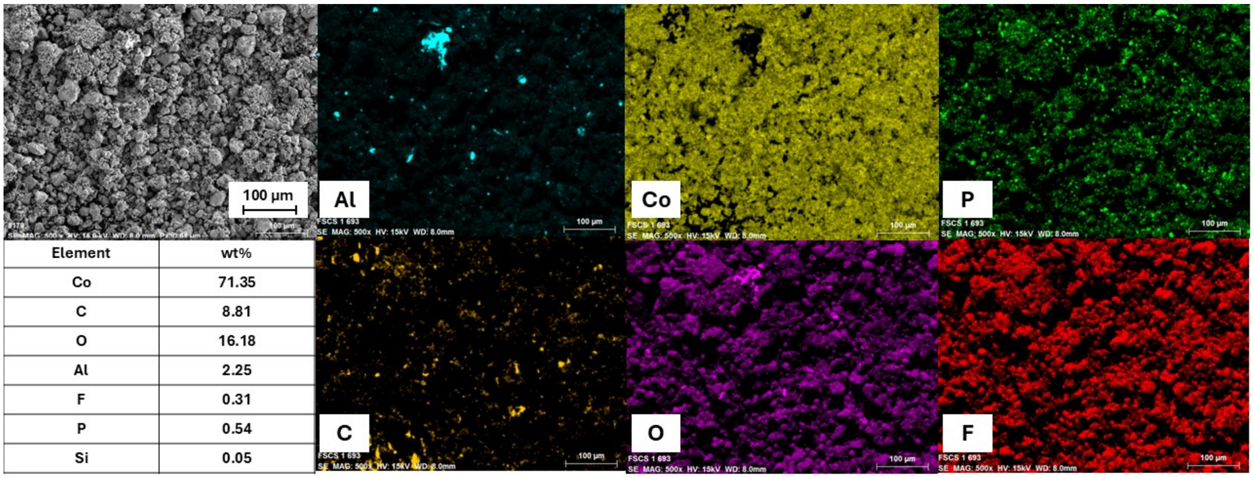

Element	wt%
Co	71.35
C	8.81
O	16.18
Al	2.25
F	0.31
P	0.54
Si	0.05

Figure S8. SEM-EDS micrographs and elemental composition for water leach residue - roasted at 500 °C, 10 wt% sucrose dosage, 60 min roasting time.

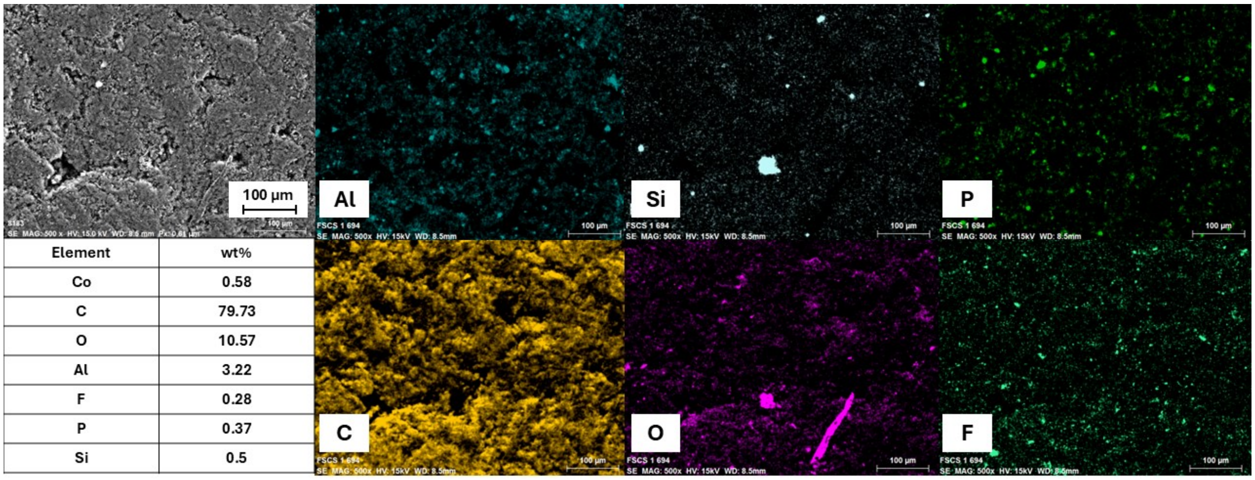

Element	wt%
Co	0.58
C	79.73
O	10.57
Al	3.22
F	0.28
P	0.37
Si	0.5

Figure S9. SEM-EDS micrographs and elemental composition for aqua regia residue - roasted at 500 °C, 10 wt% sucrose dosage, 60 min roasting time.

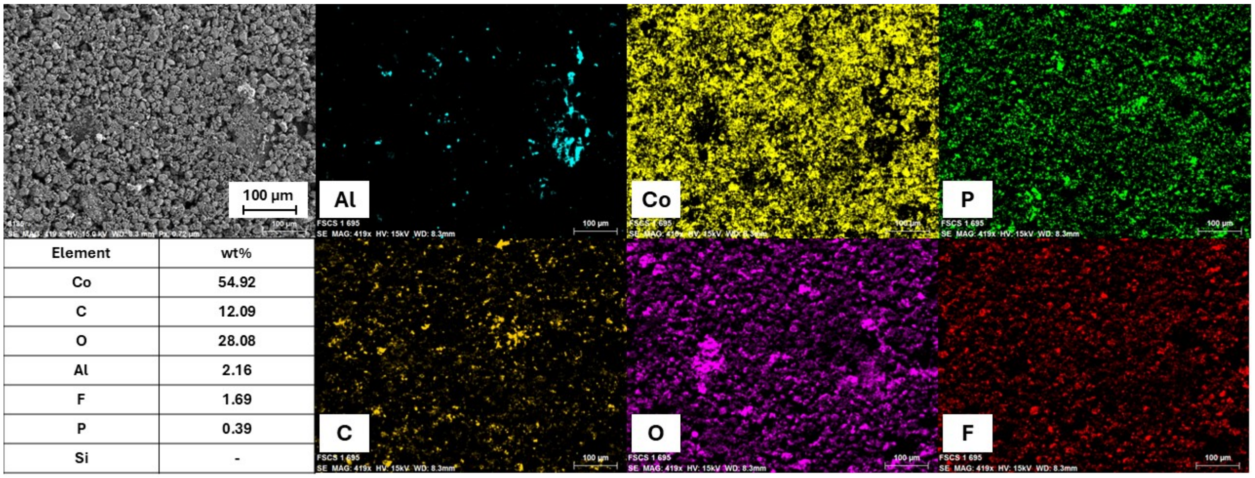

Element	wt%
Co	54.92
C	12.09
O	28.08
Al	2.16
F	1.69
P	0.39
Si	-

Figure S10. SEM-EDS micrographs and elemental composition for roasted cathode - roasted at 350 °C, 10 wt% sucrose dosage, 60 min roasting time.

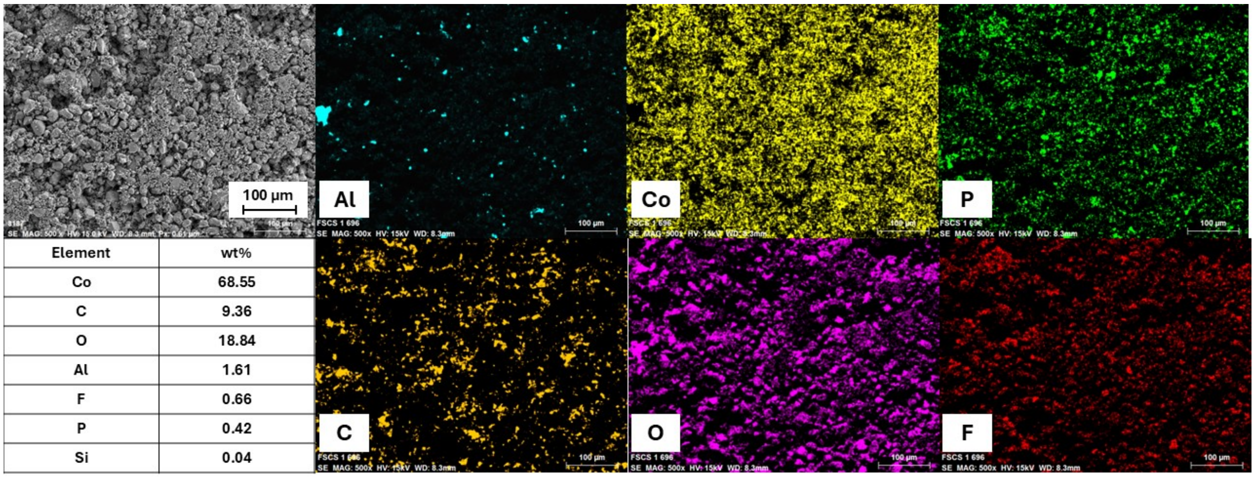

Element	wt%
Co	68.55
C	9.36
O	18.84
Al	1.61
F	0.66
P	0.42
Si	0.04

Figure S11. SEM-EDS micrographs and elemental composition for water leaching residue - roasted at 350 °C, 10 wt% sucrose dosage, 60 min roasting time.

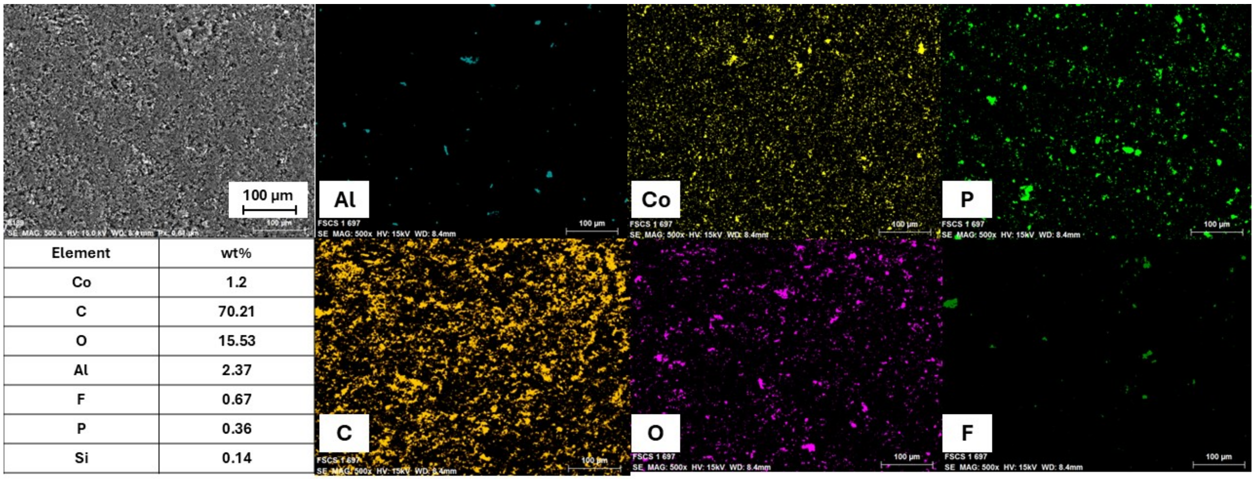

Element	wt%
Co	1.2
C	70.21
O	15.53
Al	2.37
F	0.67
P	0.36
Si	0.14

Figure S12. SEM-EDS micrographs and elemental composition for water aqua regia residue - roasted at 350 °C, 10 wt% sucrose dosage, 60 min roasting time.

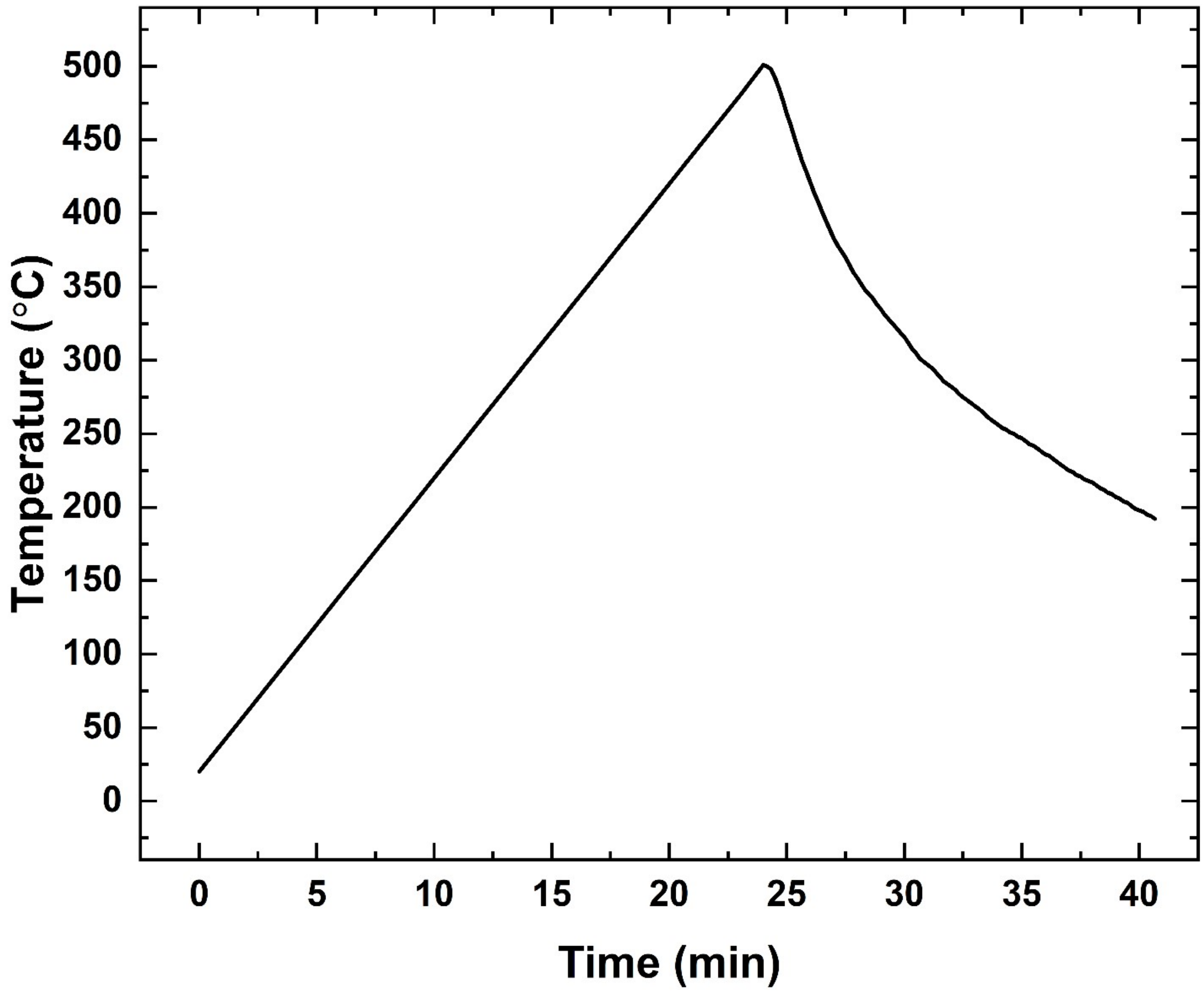

Figure S13. Measured temperature profile vs time for roasting with no holding time.

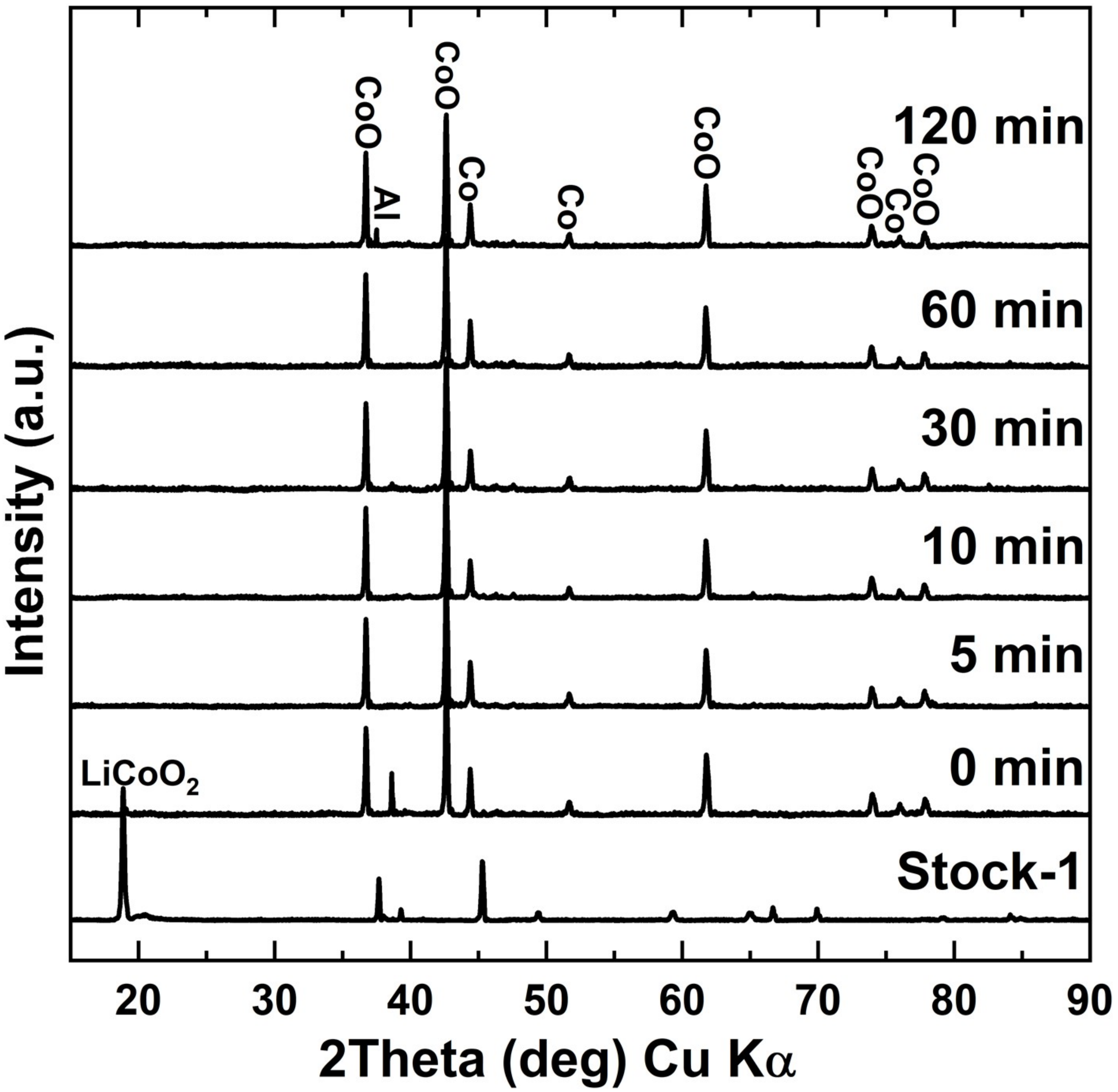

Figure S14. XRD diffractograms for aqua regia residues of sucrose dosing optimization tests. All samples were roasted at 500 °C, 15 wt% sucrose.

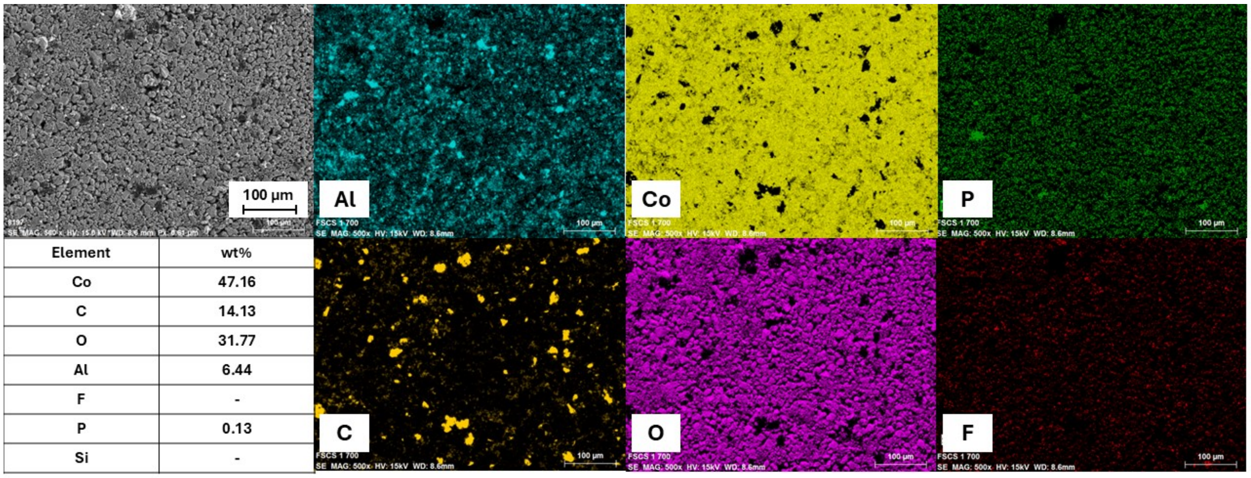

Element	wt%
Co	47.16
C	14.13
O	31.77
Al	6.44
F	-
P	0.13
Si	-

Figure S15. SEM-EDS micrographs and elemntal composition for Stock-2 after NMP dissolution pre-treatment.

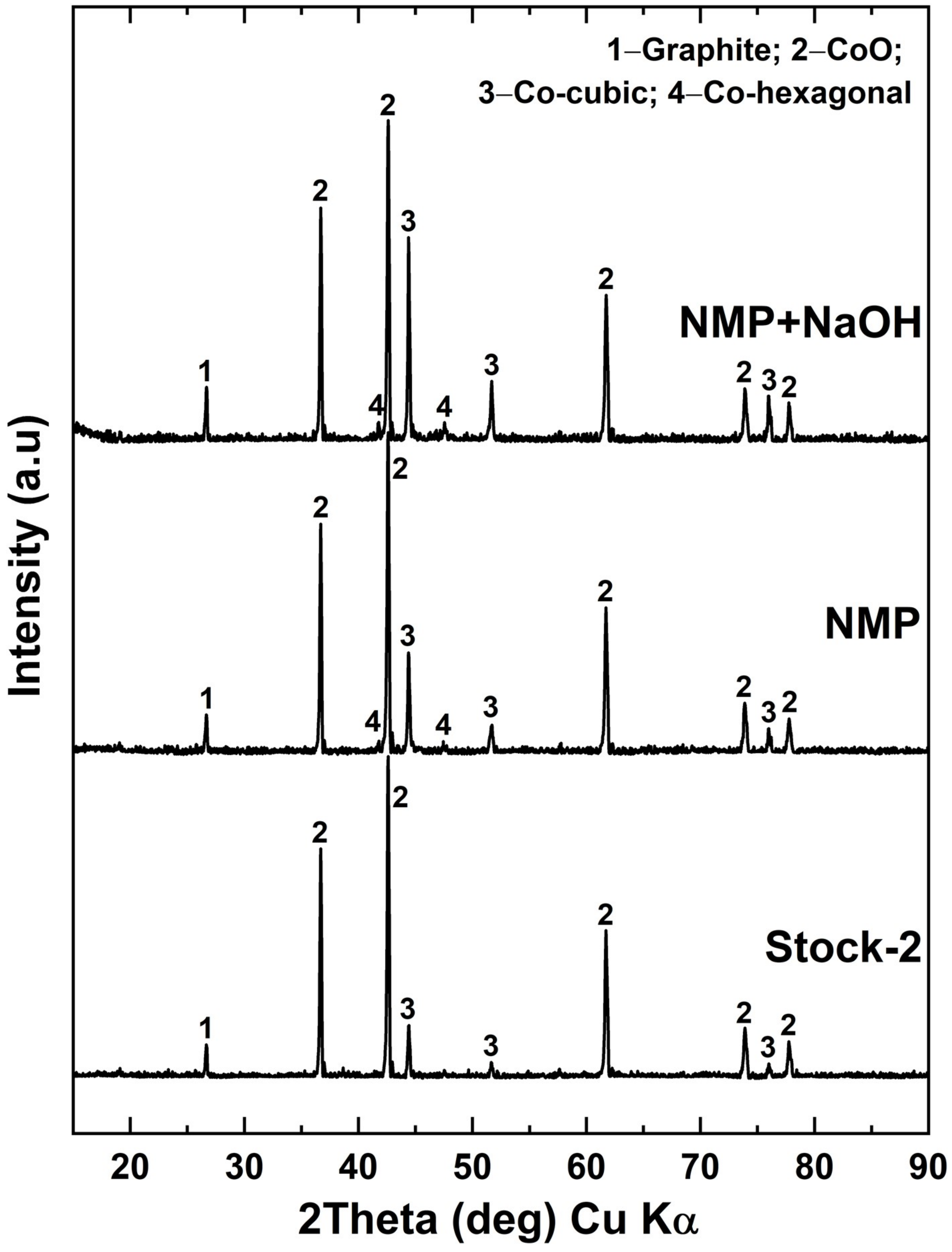

Figure S16. XRD diffractograms for water leached residues of roasted samples using different pre-treatment methods.

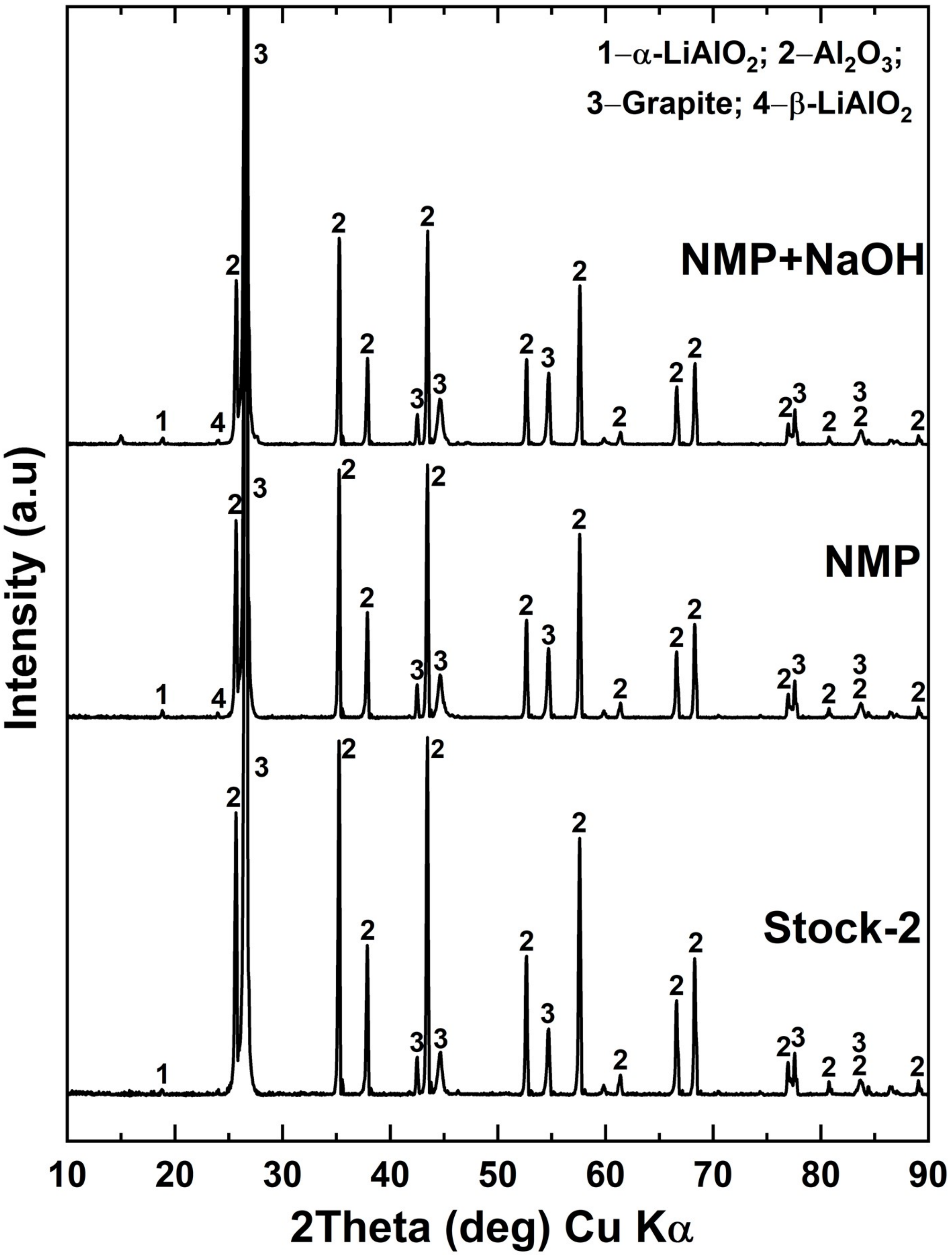

Figure S17. XRD diffractograms for aqua regia residues using different pre-treatment methods.

Table S1. Optimal roasting conditions for LCO reduction with organic reductants, with the used pre-treatment methods and the final Li leaching efficiency.

Reductant	Pre-treatment or origin of active material	Optimal roasting conditions	Li leaching efficiency
Sucrose	Manually separated cathode crushed and sieved (250 µm), NMP dissolution at 110 °C for 4 hours and 100 g/L, 4M NaOH dissolution at 60 °C for 4 hours and 100 g/L.	500 °C, 15 wt% sucrose, 60 minutes holding time.	94.5%
Pine sawdust[1]	Vacuum-dried at 373K (100 °C) for 12h, manually separated cathode electrode, electrode vacuum pyrolyzed at 723K (450 °C) for 1 hour, CAM manually peeled off from Al current collector.	873K (600 °C), Pine sawdust 1:2 LCO mass ratio, holding time 2 hours.	94.3%
Coal (lignite)[2]	Manually separated cathode, cathode crushed and sieved with a 80 mesh screen, underflow soaked in NMP.	800 °C, coal 1:3 LCO cathode, 10 min holding time.	88.7%
Glucose[3]	Discharged in saturated NaCl solution, manually separated cathode, cathode foils treated with NMP for 120 min at 90 °C, cathode active material calcined at 700 °C for 120 min.	550 °C, 10 wt% glucose, 60 min holding time.	97.0%
Corn stalk[4]	LCO peeled from Al.	600 °C,Corn stalk 0.4:1 LCO, 1 hour holdng time.	99.8%
Waste coffee[5]	Vacuum dried at 100 °C for 12 hours, manually dismantled, 1 hour pyrolysis at 450 °C, cathode active material manually peeled from Al current collector.	600 °C, coffee 1:5 LCO, 1.5 hour holding time.	89.2%
Separator (Polyethylene)[6]	Washed in deionized water, dried at 60 °C for 12 hours. Cathode manually removed and washed with ethanol and double-distilled water, roasting at 350 °C for 2 hours under vacuum to separate cathode active material from Al foil.	500 °C, PE 0.1:1 LCO, holding time 2 hours.	93.2%
Polyvinyl chloride[7]	Battery-grade raw LCO	450 °C, PVC 1:1 LCO, 1 hour holding time.	92.5%
Polycarbonate[8]	Battery-grade raw LCO	500 °C, PC 3:1 LCO, 1 hour holding time.	98.3%
Polyethylene terephthalate[8]	Battery-grade raw LCO	500 °C, PET 3:1 LCO, 3 hour holding time.	92.9%
Polystyrene[8]	Battery-grade raw LCO	500 °C, PS 3:1 LCO, 3 hour holding time.	81.5%

Polycarbonate[8]	Battery-grade raw LCO	500 °C, PC 10:1 LCO, 5 hour holding time.	99.98%

References

1 F. Zhou, X. Qu, Y. Wu, J. Zhao, S. Gao, D. Wang and H. Yin, ACS Sustain. Chem. Eng., 2022, **10**, 1287–1297, DOI: 10.1021/acssuschemeng.1c07424.

2 Y. Lai, X. Zhu, J. Li, Q. Peng, S. Hu, A. Xia, Y. Huang, Q. Liao and X. Zhu, Waste Manag., 2022, **148**, 12–21, DOI: 10.1016/j.wasman.2022.05.017.

3 B. Zhang, Y. Xu, B. Makuza, F. Zhu, H. Wang, N. Hong, Z. Long, W. Deng, G. Zou, H. Hou and X. Ji, Chem. Eng. J., 2023, **452**, 139258, DOI: 10.1016/j.cej.2022.139258.

4 X. Chen, Y. Wang, S. Li, Y. Jiang, Y. Cao and X. Ma, Chem. Eng. J., 2022, **434**, 134542, DOI: 10.1016/j.cej.2022.134542.

5 M. A. Hasan, R. Hossain and V. Sahajwalla, Green Chem., 2024, **27**, 1073–1088, DOI: 10.1039/d4gc05048g.

6 W. Hou, X. Huang, R. Tang, Y. Min, Q. Xu, Z. Hu and P. Shi, Waste Manag., 2023, **155**, 129–136, DOI: 10.1016/j.wasman.2022.11.002.

7 Z. Meng, W. Huang, L. Gao, J. Dai, X. Lu, J. Liu, H. Huang, K. Shih and Y. Tang, ACS Sustain. Chem. Eng., 2022, **10**, 12329–12341, DOI: 10.1021/acssuschemeng.2c03616.

8 B. Qiu, M. Liu, X. Qu, B. Zhang, H. Xie, D. Wang, L. Y. S. Lee and H. Yin, Environ. Sci. Technol., 2023, **57**, 7599–7611, DOI: 10.1021/acs.est.2c09816.
